# From Louvain to Leiden: guaranteeing well-connected communities

**DOI:** 10.1038/s41598-019-41695-z

**Published:** 2019-03-26

**Authors:** V. A. Traag, L. Waltman, N. J. van Eck

**Affiliations:** Centre for Science and Technology Studies, Leiden University, Leiden, The Netherlands

## Abstract

Community detection is often used to understand the structure of large and complex networks. One of the most popular algorithms for uncovering community structure is the so-called Louvain algorithm. We show that this algorithm has a major defect that largely went unnoticed until now: the Louvain algorithm may yield arbitrarily badly connected communities. In the worst case, communities may even be disconnected, especially when running the algorithm iteratively. In our experimental analysis, we observe that up to 25% of the communities are badly connected and up to 16% are disconnected. To address this problem, we introduce the Leiden algorithm. We prove that the Leiden algorithm yields communities that are guaranteed to be connected. In addition, we prove that, when the Leiden algorithm is applied iteratively, it converges to a partition in which all subsets of all communities are locally optimally assigned. Furthermore, by relying on a fast local move approach, the Leiden algorithm runs faster than the Louvain algorithm. We demonstrate the performance of the Leiden algorithm for several benchmark and real-world networks. We find that the Leiden algorithm is faster than the Louvain algorithm and uncovers better partitions, in addition to providing explicit guarantees.

## Introduction

In many complex networks, nodes cluster and form relatively dense groups—often called communities^1,2^. Such a modular structure is usually not known beforehand. Detecting communities in a network is therefore an important problem. One of the best-known methods for community detection is called modularity^3^. This method tries to maximise the difference between the actual number of edges in a community and the expected number of such edges. We denote by *e*_*c*_ the actual number of edges in community *c*. The expected number of edges can be expressed as $\frac{{K}_{c}^{2}}{2m}$, where *K*_*c*_ is the sum of the degrees of the nodes in community *c* and *m* is the total number of edges in the network. This way of defining the expected number of edges is based on the so-called configuration model. Modularity is given by

1 $$ {\mathcal H} =\frac{1}{2m}\,{\sum }_{c}({e}_{c}-{\rm{\gamma }}\frac{{K}_{c}^{2}}{2m}),$$

where γ > 0 is a resolution parameter^4^. Higher resolutions lead to more communities, while lower resolutions lead to fewer communities.

Optimising modularity is NP-hard^5^, and consequentially many heuristic algorithms have been proposed, such as hierarchical agglomeration^6^, extremal optimisation^7^, simulated annealing^4,8^ and spectral^9^ algorithms. One of the most popular algorithms to optimise modularity is the so-called Louvain algorithm^10^, named after the location of its authors. It was found to be one of the fastest and best performing algorithms in comparative analyses^11,12^, and it is one of the most-cited works in the community detection literature.

Although originally defined for modularity, the Louvain algorithm can also be used to optimise other quality functions. An alternative quality function is the Constant Potts Model (CPM)^13^, which overcomes some limitations of modularity. CPM is defined as

2 $$ {\mathcal H} ={\sum }_{c}[{e}_{c}-\gamma (\begin{array}{c}{n}_{c}\\ 2\end{array})],$$

where *n*_*c*_ is the number of nodes in community *c*. The interpretation of the resolution parameter γ is quite straightforward. The parameter functions as a sort of threshold: communities should have a density of at least γ, while the density between communities should be lower than γ. Higher resolutions lead to more communities and lower resolutions lead to fewer communities, similarly to the resolution parameter for modularity.

In this paper, we show that the Louvain algorithm has a major problem, for both modularity and CPM. The algorithm may yield arbitrarily badly connected communities, over and above the well-known issue of the resolution limit^14^. Communities may even be internally disconnected. To address this important shortcoming, we introduce a new algorithm that is faster, finds better partitions and provides explicit guarantees and bounds. The new algorithm integrates several earlier improvements, incorporating a combination of smart local move^15^, fast local move^16,17^ and random neighbour move^18^. We prove that the new algorithm is guaranteed to produce partitions in which all communities are internally connected. In addition, we prove that the algorithm converges to an asymptotically stable partition in which all subsets of all communities are locally optimally assigned. The quality of such an asymptotically stable partition provides an upper bound on the quality of an optimal partition. Finally, we demonstrate the excellent performance of the algorithm for several benchmark and real-world networks. To ensure readability of the paper to the broadest possible audience, we have chosen to relegate all technical details to the Supplementary Information. The main ideas of our algorithm are explained in an intuitive way in the main text of the paper. We name our algorithm the *Leiden algorithm*, after the location of its authors.

## Louvain Algorithm

**Figure 1:**
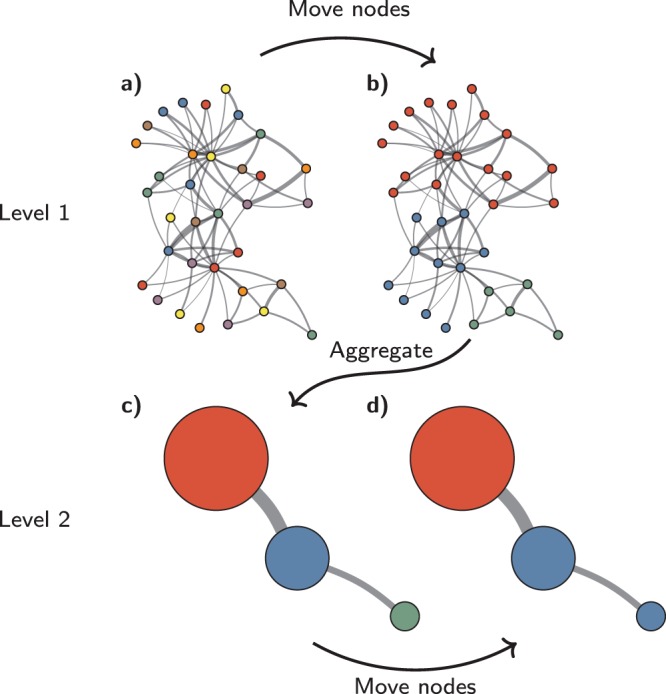
Louvain algorithm. The Louvain algorithm starts from a singleton partition in which each node is in its own community (**a**). The algorithm moves individual nodes from one community to another to find a partition (**b**). Based on this partition, an aggregate network is created (**c**). The algorithm then moves individual nodes in the aggregate network (**d**). These steps are repeated until the quality cannot be increased further.

The Louvain algorithm^10^ is very simple and elegant. The algorithm optimises a quality function such as modularity or CPM in two elementary phases: (1) local moving of nodes; and (2) aggregation of the network. In the local moving phase, individual nodes are moved to the community that yields the largest increase in the quality function. In the aggregation phase, an aggregate network is created based on the partition obtained in the local moving phase. Each community in this partition becomes a node in the aggregate network. The two phases are repeated until the quality function cannot be increased further. The Louvain algorithm is illustrated in Fig. 1 and summarised in pseudo-code in Algorithm A.1 in Section A of the Supplementary Information.

Usually, the Louvain algorithm starts from a singleton partition, in which each node is in its own community. However, it is also possible to start the algorithm from a different partition^15^. In particular, in an attempt to find better partitions, multiple consecutive iterations of the algorithm can be performed, using the partition identified in one iteration as starting point for the next iteration.

### Badly connected communities

We now show that the Louvain algorithm may find arbitrarily badly connected communities. In particular, we show that Louvain may identify communities that are internally disconnected. That is, one part of such an internally disconnected community can reach another part only through a path going outside the community. Importantly, the problem of disconnected communities is not just a theoretical curiosity. As we will demonstrate in our experimental analysis, the problem occurs frequently in practice when using the Louvain algorithm. Perhaps surprisingly, iterating the algorithm aggravates the problem, even though it does increase the quality function.

In the Louvain algorithm, a node may be moved to a different community while it may have acted as a bridge between different components of its old community. Removing such a node from its old community disconnects the old community. One may expect that other nodes in the old community will then also be moved to other communities. However, this is not necessarily the case, as the other nodes may still be sufficiently strongly connected to their community, despite the fact that the community has become disconnected.

**Figure 2:**
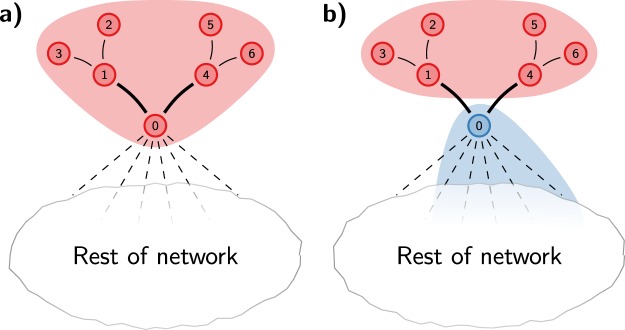
Disconnected community. Consider the partition shown in (**a**). When node 0 is moved to a different community, the red community becomes internally disconnected, as shown in (**b**). However, nodes 1–6 are still locally optimally assigned, and therefore these nodes will stay in the red community.

To elucidate the problem, we consider the example illustrated in Fig. 2. The numerical details of the example can be found in Section B of the Supplementary Information. The thick edges in Fig. 2 represent stronger connections, while the other edges represent weaker connections. At some point, the Louvain algorithm may end up in the community structure shown in Fig. 2(a). Nodes 0–6 are in the same community. Nodes 1–6 have connections only within this community, whereas node 0 also has many external connections. The algorithm continues to move nodes in the rest of the network. At some point, node 0 is considered for moving. When a sufficient number of neighbours of node 0 have formed a community in the rest of the network, it may be optimal to move node 0 to this community, thus creating the situation depicted in Fig. 2(b). In this new situation, nodes 2, 3, 5 and 6 have only internal connections. These nodes are therefore optimally assigned to their current community. On the other hand, after node 0 has been moved to a different community, nodes 1 and 4 have not only internal but also external connections. Nevertheless, depending on the relative strengths of the different connections, these nodes may still be optimally assigned to their current community. In that case, nodes 1–6 are all locally optimally assigned, despite the fact that their community has become disconnected. Clearly, it would be better to split up the community. Nodes 1–3 should form a community and nodes 4–6 should form another community. However, the Louvain algorithm does not consider this possibility, since it considers only individual node movements. Moreover, when no more nodes can be moved, the algorithm will aggregate the network. When a disconnected community has become a node in an aggregate network, there are no more possibilities to split up the community. Hence, the community remains disconnected, unless it is merged with another community that happens to act as a bridge.

Obviously, this is a worst case example, showing that disconnected communities may be identified by the Louvain algorithm. More subtle problems may occur as well, causing Louvain to find communities that are connected, but only in a very weak sense. Hence, in general, Louvain may find arbitrarily badly connected communities.

This problem is different from the well-known issue of the resolution limit of modularity^14^. Due to the resolution limit, modularity may cause smaller communities to be clustered into larger communities. In other words, modularity may “hide” smaller communities and may yield communities containing significant substructure. CPM does not suffer from this issue^13^. Nevertheless, when CPM is used as the quality function, the Louvain algorithm may still find arbitrarily badly connected communities. Hence, the problem of Louvain outlined above is independent from the issue of the resolution limit. In the case of modularity, communities may have significant substructure both because of the resolution limit and because of the shortcomings of Louvain.

In fact, although it may seem that the Louvain algorithm does a good job at finding high quality partitions, in its standard form the algorithm provides only one guarantee: the algorithm yields partitions for which it is guaranteed that no communities can be merged. In other words, communities are guaranteed to be well separated. Somewhat stronger guarantees can be obtained by iterating the algorithm, using the partition obtained in one iteration of the algorithm as starting point for the next iteration. When iterating Louvain, the quality of the partitions will keep increasing until the algorithm is unable to make any further improvements. At this point, it is guaranteed that each individual node is optimally assigned. In this iterative scheme, Louvain provides two guarantees: (1) no communities can be merged and (2) no nodes can be moved.

Contrary to what might be expected, iterating the Louvain algorithm aggravates the problem of badly connected communities, as we will also see in our experimental analysis. This is not too difficult to explain. After the first iteration of the Louvain algorithm, some partition has been obtained. In the first step of the next iteration, Louvain will again move individual nodes in the network. Some of these nodes may very well act as bridges, similarly to node 0 in the above example. By moving these nodes, Louvain creates badly connected communities. Moreover, Louvain has no mechanism for fixing these communities. Iterating the Louvain algorithm can therefore be seen as a double-edged sword: it improves the partition in some way, but degrades it in another way.

The problem of disconnected communities has been observed before^19,20^, also in the context of the label propagation algorithm^21^. However, so far this problem has never been studied for the Louvain algorithm. Moreover, the deeper significance of the problem was not recognised: disconnected communities are merely the most extreme manifestation of the problem of arbitrarily badly connected communities. Trying to fix the problem by simply considering the connected components of communities^19–21^ is unsatisfactory because it addresses only the most extreme case and does not resolve the more fundamental problem. We therefore require a more principled solution, which we will introduce in the next section.

## Leiden Algorithm

**Figure 3:**
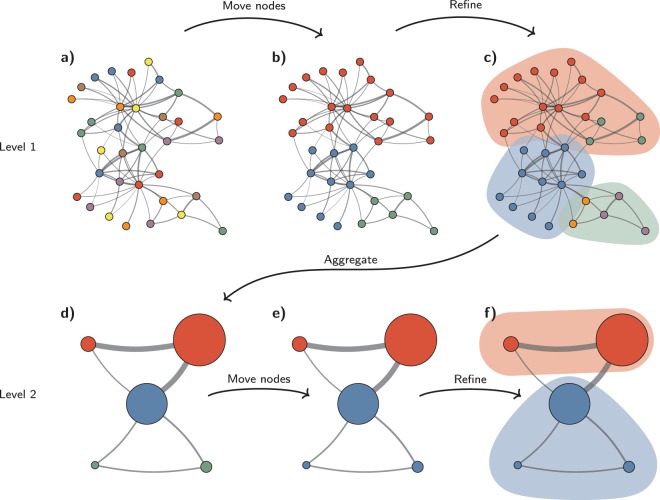
Leiden algorithm. The Leiden algorithm starts from a singleton partition (**a**). The algorithm moves individual nodes from one community to another to find a partition (**b**), which is then refined (**c**). An aggregate network (**d**) is created based on the refined partition, using the non-refined partition to create an initial partition for the aggregate network. For example, the red community in (**b**) is refined into two subcommunities in (**c**), which after aggregation become two separate nodes in (**d**), both belonging to the same community. The algorithm then moves individual nodes in the aggregate network (**e**). In this case, refinement does not change the partition (**f**). These steps are repeated until no further improvements can be made.

We here introduce the Leiden algorithm, which guarantees that communities are well connected. The Leiden algorithm is partly based on the previously introduced smart local move algorithm^15^, which itself can be seen as an improvement of the Louvain algorithm. The Leiden algorithm also takes advantage of the idea of speeding up the local moving of nodes^16,17^ and the idea of moving nodes to random neighbours^18^. We consider these ideas to represent the most promising directions in which the Louvain algorithm can be improved, even though we recognise that other improvements have been suggested as well^22^. The Leiden algorithm consists of three phases: (1) local moving of nodes, (2) refinement of the partition and (3) aggregation of the network based on the refined partition, using the non-refined partition to create an initial partition for the aggregate network. The Leiden algorithm is considerably more complex than the Louvain algorithm. Figure 3 provides an illustration of the algorithm. The algorithm is described in pseudo-code in Algorithm A.2 in Section A of the Supplementary Information.

In the Louvain algorithm, an aggregate network is created based on the partition ${\mathscr{P}}$ resulting from the local moving phase. The idea of the refinement phase in the Leiden algorithm is to identify a partition ${{\mathscr{P}}}_{{\rm{refined}}}$ that is a refinement of ${\mathscr{P}}$. Communities in ${\mathscr{P}}$ may be split into multiple subcommunities in ${{\mathscr{P}}}_{{\rm{refined}}}$. The aggregate network is created based on the partition ${{\mathscr{P}}}_{{\rm{refined}}}$. However, the initial partition for the aggregate network is based on *P*, just like in the Louvain algorithm. By creating the aggregate network based on ${{\mathscr{P}}}_{{\rm{refined}}}$ rather than *P*, the Leiden algorithm has more room for identifying high-quality partitions. In fact, by implementing the refinement phase in the right way, several attractive guarantees can be given for partitions produced by the Leiden algorithm.

The refined partition ${{\mathscr{P}}}_{{\rm{refined}}}$ is obtained as follows. Initially, ${{\mathscr{P}}}_{{\rm{refined}}}$ is set to a singleton partition, in which each node is in its own community. The algorithm then locally merges nodes in ${{\mathscr{P}}}_{{\rm{refined}}}$: nodes that are on their own in a community in ${{\mathscr{P}}}_{{\rm{refined}}}$ can be merged with a different community. Importantly, mergers are performed only within each community of the partition ${\mathscr{P}}$. In addition, a node is merged with a community in ${{\mathscr{P}}}_{{\rm{refined}}}$ only if both are sufficiently well connected to their community in ${\mathscr{P}}$. After the refinement phase is concluded, communities in ${\mathscr{P}}$ often will have been split into multiple communities in ${{\mathscr{P}}}_{{\rm{refined}}}$, but not always.

In the refinement phase, nodes are not necessarily greedily merged with the community that yields the largest increase in the quality function. Instead, a node may be merged with any community for which the quality function increases. The community with which a node is merged is selected randomly^18^. The larger the increase in the quality function, the more likely a community is to be selected. The degree of randomness in the selection of a community is determined by a parameter *θ* > 0. Randomness in the selection of a community allows the partition space to be explored more broadly. Node mergers that cause the quality function to decrease are not considered. This contrasts with optimisation algorithms such as simulated annealing, which do allow the quality function to decrease^4,8^. Such algorithms are rather slow, making them ineffective for large networks. Excluding node mergers that decrease the quality function makes the refinement phase more efficient. As we prove in Section C1 of the Supplementary Information, even when node mergers that decrease the quality function are excluded, the optimal partition of a set of nodes can still be uncovered. This is not the case when nodes are greedily merged with the community that yields the largest increase in the quality function. In that case, some optimal partitions cannot be found, as we show in Section C2 of the Supplementary Information.

Another important difference between the Leiden algorithm and the Louvain algorithm is the implementation of the local moving phase. Unlike the Louvain algorithm, the Leiden algorithm uses a fast local move procedure in this phase. Louvain keeps visiting all nodes in a network until there are no more node movements that increase the quality function. In doing so, Louvain keeps visiting nodes that cannot be moved to a different community. In the fast local move procedure in the Leiden algorithm, only nodes whose neighbourhood has changed are visited. This is similar to ideas proposed recently as “pruning”^16^ and in a slightly different form as “prioritisation”^17^. The fast local move procedure can be summarised as follows. We start by initialising a queue with all nodes in the network. The nodes are added to the queue in a random order. We then remove the first node from the front of the queue and we determine whether the quality function can be increased by moving this node from its current community to a different one. If we move the node to a different community, we add to the rear of the queue all neighbours of the node that do not belong to the node’s new community and that are not yet in the queue. We keep removing nodes from the front of the queue, possibly moving these nodes to a different community. This continues until the queue is empty. For a full specification of the fast local move procedure, we refer to the pseudo-code of the Leiden algorithm in Algorithm A.2 in Section A of the Supplementary Information. Using the fast local move procedure, the first visit to all nodes in a network in the Leiden algorithm is the same as in the Louvain algorithm. However, after all nodes have been visited once, Leiden visits only nodes whose neighbourhood has changed, whereas Louvain keeps visiting all nodes in the network. In this way, Leiden implements the local moving phase more efficiently than Louvain.

### Guarantees

We now consider the guarantees provided by the Leiden algorithm. The algorithm is run iteratively, using the partition identified in one iteration as starting point for the next iteration. We can guarantee a number of properties of the partitions found by the Leiden algorithm at various stages of the iterative process. Below we offer an intuitive explanation of these properties. We provide the full definitions of the properties as well as the mathematical proofs in Section D of the Supplementary Information.

After each iteration of the Leiden algorithm, it is guaranteed that:

1. All communities are γ-separated.
2. All communities are γ-connected.
  In these properties, γ refers to the resolution parameter in the quality function that is optimised, which can be either modularity or CPM. The property of γ-separation is also guaranteed by the Louvain algorithm. It states that there are no communities that can be merged. The property of γ-connectivity is a slightly stronger variant of ordinary connectivity. As discussed earlier, the Louvain algorithm does not guarantee connectivity. It therefore does not guarantee γ-connectivity either.
  An iteration of the Leiden algorithm in which the partition does not change is called a stable iteration. After a stable iteration of the Leiden algorithm, it is guaranteed that:
3. All nodes are locally optimally assigned.
4. All communities are subpartition γ-dense.
  Node optimality is also guaranteed after a stable iteration of the Louvain algorithm. It means that there are no individual nodes that can be moved to a different community. Subpartition γ-density is not guaranteed by the Louvain algorithm. A community is subpartition γ-dense if it can be partitioned into two parts such that: (1) the two parts are well connected to each other; (2) neither part can be separated from its community; and (3) each part is also subpartition γ-dense itself. Subpartition γ-density does not imply that individual nodes are locally optimally assigned. It only implies that individual nodes are well connected to their community.
  In the case of the Louvain algorithm, after a stable iteration, all subsequent iterations will be stable as well. Hence, no further improvements can be made after a stable iteration of the Louvain algorithm. This contrasts with the Leiden algorithm. After a stable iteration of the Leiden algorithm, the algorithm may still be able to make further improvements in later iterations. In fact, when we keep iterating the Leiden algorithm, it will converge to a partition for which it is guaranteed that:
5. All communities are uniformly γ-dense.
6. All communities are subset optimal.

A community is uniformly γ-dense if there are no subsets of the community that can be separated from the community. Uniform γ-density means that no matter how a community is partitioned into two parts, the two parts will always be well connected to each other. Furthermore, if all communities in a partition are uniformly γ-dense, the quality of the partition is not too far from optimal, as shown in Section E of the Supplementary Information. A community is subset optimal if all subsets of the community are locally optimally assigned. That is, no subset can be moved to a different community. Subset optimality is the strongest guarantee that is provided by the Leiden algorithm. It implies uniform γ-density and all the other above-mentioned properties.

An overview of the various guarantees is presented in Table 1.

**Table 1:** Overview of the guarantees provided by the Louvain algorithm and the Leiden algorithm.

		Louvain	Leiden
Each iteration	γ-separation	✓	✓
	γ-connectivity		✓
Stable iteration	Node optimality	✓	✓
	Subpartition γ-density		✓
Asymptotic	Uniform γ-density		✓
	Subset optimality		✓

## Experimental Analysis

In the previous section, we showed that the Leiden algorithm guarantees a number of properties of the partitions uncovered at different stages of the algorithm. We also suggested that the Leiden algorithm is faster than the Louvain algorithm, because of the fast local move approach. In this section, we analyse and compare the performance of the two algorithms in practice. (We implemented both algorithms in Java, available from https://github.com/CWTSLeiden/networkanalysis and deposited at Zenodo^23^. Additionally, we implemented a Python package, available from https://github.com/vtraag/leidenalg and deposited at Zenodo^24^). All experiments were run on a computer with 64 Intel Xeon E5-4667v3 2 GHz CPUs and 1 TB internal memory. In all experiments reported here, we used a value of 0.01 for the parameter *θ* that determines the degree of randomness in the refinement phase of the Leiden algorithm. However, values of *θ* within a range of roughly [0.0005, 0.1] all provide reasonable results, thus allowing for some, but not too much randomness. We use six empirical networks in our analysis. These are the same networks that were also studied in an earlier paper introducing the smart local move algorithm^15^. Table 2 provides an overview of the six networks. First, we show that the Louvain algorithm finds disconnected communities, and more generally, badly connected communities in the empirical networks. Second, to study the scaling of the Louvain and the Leiden algorithm, we use benchmark networks, allowing us to compare the algorithms in terms of both computational time and quality of the partitions. Finally, we compare the performance of the algorithms on the empirical networks. We find that the Leiden algorithm commonly finds partitions of higher quality in less time. The difference in computational time is especially pronounced for larger networks, with Leiden being up to 20 times faster than Louvain in empirical networks.

**Table 2:** Overview of the empirical networks and of the maximal modularity after 10 replications of 10 iterations each, both for the Louvain and for the Leiden algorithm.

	Nodes	Degree	Max. modularity	
			Louvain	Leiden
DBLP	317,080	6.6	0.8262	0.8387
Amazon	334,863	5.6	0.9301	0.9341
IMDB	374,511	80.2	0.7062	0.7069
Live Journal	3,997,962	17.4	0.7653	0.7739
Web of Science	9,811,130	21.2	0.7911	0.7951
Web UK	39,252,879	39.8	0.9796	0.9801

### Badly connected communities

We study the problem of badly connected communities when using the Louvain algorithm for several empirical networks. For each community in a partition that was uncovered by the Louvain algorithm, we determined whether it is internally connected or not. In addition, to analyse whether a community is badly connected, we ran the Leiden algorithm on the subnetwork consisting of all nodes belonging to the community. (We ensured that modularity optimisation for the subnetwork was fully consistent with modularity optimisation for the whole network^13^) The Leiden algorithm was run until a stable iteration was obtained. When the Leiden algorithm found that a community could be split into multiple subcommunities, we counted the community as badly connected. Note that if Leiden finds subcommunities, splitting up the community is guaranteed to increase modularity. Conversely, if Leiden does not find subcommunities, there is no guarantee that modularity cannot be increased by splitting up the community. Hence, by counting the number of communities that have been split up, we obtained a lower bound on the number of communities that are badly connected. The count of badly connected communities also included disconnected communities. For each network, we repeated the experiment 10 times. We used modularity with a resolution parameter of γ = 1 for the experiments.

**Figure 4:**
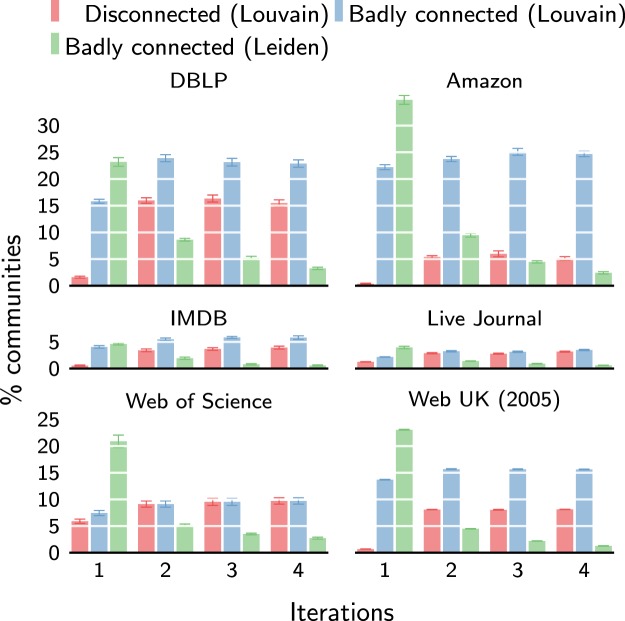
Badly connected communities. Percentage of communities found by the Louvain algorithm that are either disconnected or badly connected compared to percentage of badly connected communities found by the Leiden algorithm. Note that communities found by the Leiden algorithm are guaranteed to be connected.

As can be seen in Fig. 4, in the first iteration of the Louvain algorithm, the percentage of badly connected communities can be quite high. For the Amazon, DBLP and Web UK networks, Louvain yields on average respectively 23%, 16% and 14% badly connected communities. The percentage of disconnected communities is more limited, usually around 1%. However, in the case of the Web of Science network, more than 5% of the communities are disconnected in the first iteration.

Later iterations of the Louvain algorithm only aggravate the problem of disconnected communities, even though the quality function (i.e. modularity) increases. The second iteration of Louvain shows a large increase in the percentage of disconnected communities. In subsequent iterations, the percentage of disconnected communities remains fairly stable. The increase in the percentage of disconnected communities is relatively limited for the Live Journal and Web of Science networks. Other networks show an almost tenfold increase in the percentage of disconnected communities. The percentage of disconnected communities even jumps to 16% for the DBLP network. The percentage of badly connected communities is less affected by the number of iterations of the Louvain algorithm. Presumably, many of the badly connected communities in the first iteration of Louvain become disconnected in the second iteration. Indeed, the percentage of disconnected communities becomes more comparable to the percentage of badly connected communities in later iterations. Nonetheless, some networks still show large differences. For example, after four iterations, the Web UK network has 8% disconnected communities, but twice as many badly connected communities. Even worse, the Amazon network has 5% disconnected communities, but 25% badly connected communities.

The above results shows that the problem of disconnected and badly connected communities is quite pervasive in practice. Because the percentage of disconnected communities in the first iteration of the Louvain algorithm usually seems to be relatively low, the problem may have escaped attention from users of the algorithm. However, focussing only on disconnected communities masks the more fundamental issue: Louvain finds arbitrarily badly connected communities. The high percentage of badly connected communities attests to this. Besides being pervasive, the problem is also sizeable. In the worst case, almost a quarter of the communities are badly connected. This may have serious consequences for analyses based on the resulting partitions. For example, nodes in a community in biological or neurological networks are often assumed to share similar functions or behaviour^25^. However, if communities are badly connected, this may lead to incorrect attributions of shared functionality. Similarly, in citation networks, such as the Web of Science network, nodes in a community are usually considered to share a common topic^26,27^. Again, if communities are badly connected, this may lead to incorrect inferences of topics, which will affect bibliometric analyses relying on the inferred topics. In short, the problem of badly connected communities has important practical consequences.

The Leiden algorithm has been specifically designed to address the problem of badly connected communities. Figure 4 shows how well it does compared to the Louvain algorithm. The Leiden algorithm guarantees all communities to be connected, but it may yield badly connected communities. In terms of the percentage of badly connected communities in the first iteration, Leiden performs even worse than Louvain, as can be seen in Fig. 4. Crucially, however, the percentage of badly connected communities decreases with each iteration of the Leiden algorithm. Starting from the second iteration, Leiden outperformed Louvain in terms of the percentage of badly connected communities. In fact, if we keep iterating the Leiden algorithm, it will converge to a partition without any badly connected communities, as discussed earlier. Hence, the Leiden algorithm effectively addresses the problem of badly connected communities.

### Benchmark networks

To study the scaling of the Louvain and the Leiden algorithm, we rely on a variant of a well-known approach for constructing benchmark networks^28^. We generated benchmark networks in the following way. First, we created a specified number of nodes and we assigned each node to a community. Communities were all of equal size. A community size of 50 nodes was used for the results presented below, but larger community sizes yielded qualitatively similar results. We then created a certain number of edges such that a specified average degree $\langle k\rangle$ was obtained. For the results reported below, the average degree was set to $\langle k\rangle =10$. Edges were created in such a way that an edge fell between two communities with a probability *μ* and within a community with a probability 1−*μ*. We applied the Louvain and the Leiden algorithm to exactly the same networks, using the same seed for the random number generator. For both algorithms, 10 iterations were performed. We used the CPM quality function. The value of the resolution parameter was determined based on the so-called mixing parameter *μ*^13^. We generated networks with *n* = 10^3^ to *n* = 10^7^ nodes. For each set of parameters, we repeated the experiment 10 times. Below, the quality of a partition is reported as $\frac{ {\mathcal H} }{2m}$, where *H* is defined in Eq. (2) and *m* is the number of edges.

**Figure 5:**
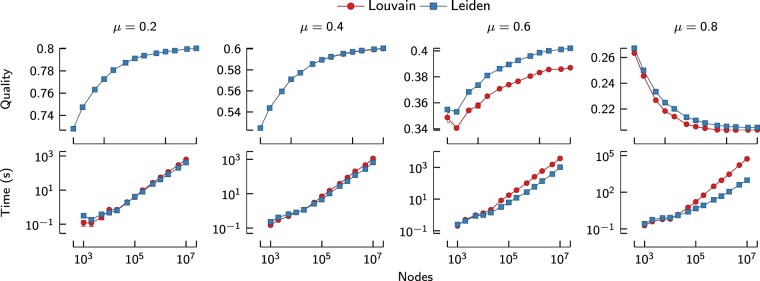
Scaling of benchmark results for network size. Speed and quality of the Louvain and the Leiden algorithm for benchmark networks of increasing size (two iterations). For larger networks and higher values of *μ*, Louvain is much slower than Leiden. For higher values of *μ*, Leiden finds better partitions than Louvain.

As shown in Fig. 5, for lower values of *μ* the partition is well defined, and neither the Louvain nor the Leiden algorithm has a problem in determining the correct partition in only two iterations. Hence, for lower values of *μ*, the difference in quality is negligible. However, as *μ* increases, the Leiden algorithm starts to outperform the Louvain algorithm. The differences are not very large, which is probably because both algorithms find partitions for which the quality is close to optimal, related to the issue of the degeneracy of quality functions^29^.

The Leiden algorithm is clearly faster than the Louvain algorithm. For lower values of *μ*, the correct partition is easy to find and Leiden is only about twice as fast as Louvain. However, for higher values of *μ*, Leiden becomes orders of magnitude faster than Louvain, reaching 10–100 times faster runtimes for the largest networks. As can be seen in Fig. 7, whereas Louvain becomes much slower for more difficult partitions, Leiden is much less affected by the difficulty of the partition.

**Figure 6:**
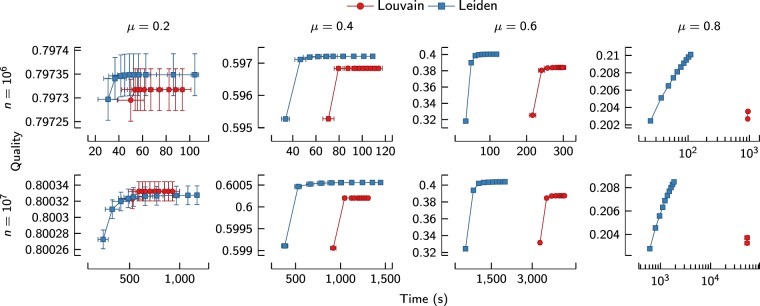
Runtime versus quality for benchmark networks. Speed and quality for the first 10 iterations of the Louvain and the Leiden algorithm for benchmark networks (*n* = 10^6^ and *n* = 10^7^). The horizontal axis indicates the cumulative time taken to obtain the quality indicated on the vertical axis. Each point corresponds to a certain iteration of an algorithm, with results averaged over 10 experiments. In general, Leiden is both faster than Louvain and finds better partitions.

Figure 6 presents total runtime versus quality for all iterations of the Louvain and the Leiden algorithm. As can be seen in the figure, Louvain quickly reaches a state in which it is unable to find better partitions. On the other hand, Leiden keeps finding better partitions, especially for higher values of *μ*, for which it is more difficult to identify good partitions. A number of iterations of the Leiden algorithm can be performed before the Louvain algorithm has finished its first iteration. Later iterations of the Louvain algorithm are very fast, but this is only because the partition remains the same. With one exception (*μ* = 0.2 and *n* = 10^7^), all results in Fig. 6 show that Leiden outperforms Louvain in terms of both computational time and quality of the partitions.

**Figure 7:**
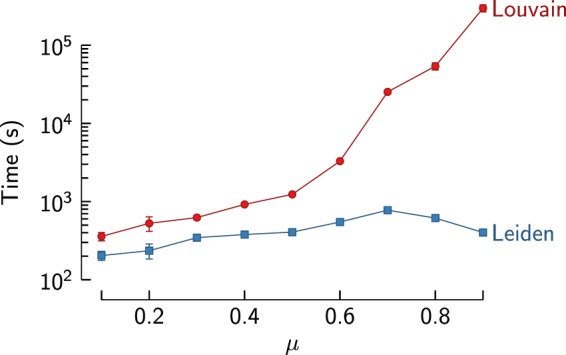
Scaling of benchmark results for difficulty of the partition. Speed of the first iteration of the Louvain and the Leiden algorithm for benchmark networks with increasingly difficult partitions (*n* = 10^7^). In the most difficult case (*μ* = 0.9), Louvain requires almost 2.5 days, while Leiden needs fewer than 10 minutes.

### Empirical networks

Analyses based on benchmark networks have only a limited value because these networks are not representative of empirical real-world networks. In particular, benchmark networks have a rather simple structure. Empirical networks show a much richer and more complex structure. We now compare how the Leiden and the Louvain algorithm perform for the six empirical networks listed in Table 2. Our analysis is based on modularity with resolution parameter γ = 1. For each network, Table 2 reports the maximal modularity obtained using the Louvain and the Leiden algorithm.

**Figure 8:**
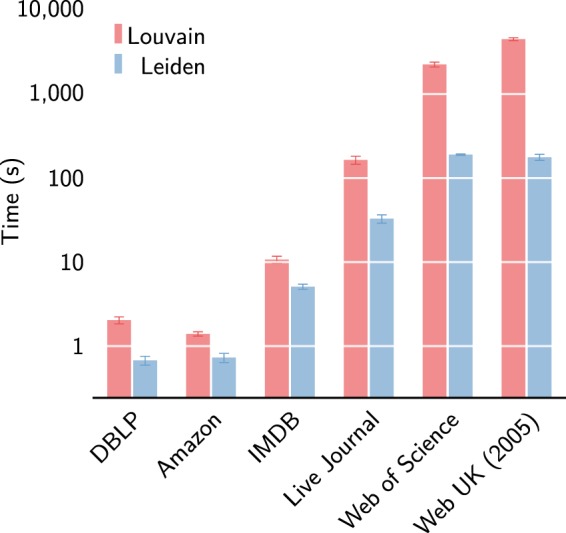
First iteration runtime for empirical networks. Speed of the first iteration of the Louvain and the Leiden algorithm for six empirical networks. Leiden is faster than Louvain especially for larger networks.

**Figure 9:**
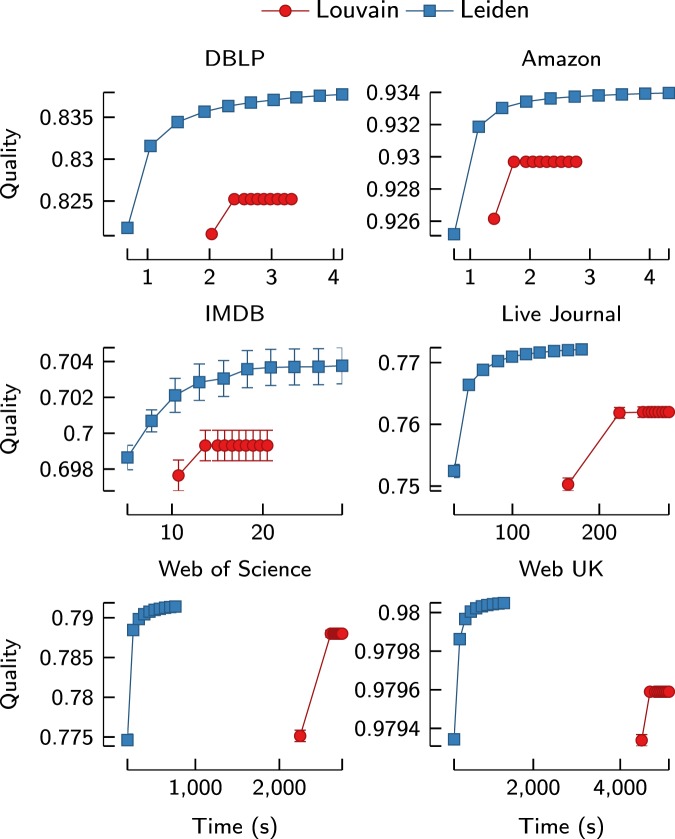
Runtime versus quality for empirical networks. Speed and quality for the first 10 iterations of the Louvain and the Leiden algorithm for six empirical networks. The horizontal axis indicates the cumulative time taken to obtain the quality indicated on the vertical axis. Each point corresponds to a certain iteration of an algorithm, with results averaged over 10 experiments. Leiden is both faster than Louvain and finds better partitions.

As can be seen in Fig. 8, the Leiden algorithm is significantly faster than the Louvain algorithm also in empirical networks. In the first iteration, Leiden is roughly 2–20 times faster than Louvain. The speed difference is especially large for larger networks. This is similar to what we have seen for benchmark networks. For the Amazon and IMDB networks, the first iteration of the Leiden algorithm is only about 1.6 times faster than the first iteration of the Louvain algorithm. However, Leiden is more than 7 times faster for the Live Journal network, more than 11 times faster for the Web of Science network and more than 20 times faster for the Web UK network. In fact, for the Web of Science and Web UK networks, Fig. 9 shows that more than 10 iterations of the Leiden algorithm can be performed before the Louvain algorithm has finished its first iteration.

As shown in Fig. 9, the Leiden algorithm also performs better than the Louvain algorithm in terms of the quality of the partitions that are obtained. For all networks, Leiden identifies substantially better partitions than Louvain. Louvain quickly converges to a partition and is then unable to make further improvements. In contrast, Leiden keeps finding better partitions in each iteration.

**Figure 10:**
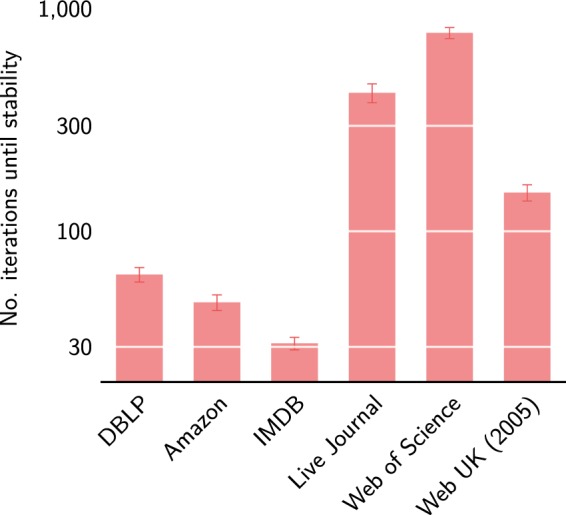
Number of iterations until stability. Number of iterations before the Leiden algorithm has reached a stable iteration for six empirical networks. In a stable iteration, the partition is guaranteed to be node optimal and subpartition γ-dense.

The quality improvement realised by the Leiden algorithm relative to the Louvain algorithm is larger for empirical networks than for benchmark networks. Hence, the complex structure of empirical networks creates an even stronger need for the use of the Leiden algorithm. Leiden keeps finding better partitions for empirical networks also after the first 10 iterations of the algorithm. This contrasts to benchmark networks, for which Leiden often converges after a few iterations. For empirical networks, it may take quite some time before the Leiden algorithm reaches its first stable iteration. As can be seen in Fig. 10, for the IMDB and Amazon networks, Leiden reaches a stable iteration relatively quickly, presumably because these networks have a fairly simple community structure. The DBLP network is somewhat more challenging, requiring almost 80 iterations on average to reach a stable iteration. The Web of Science network is the most difficult one. For this network, Leiden requires over 750 iterations on average to reach a stable iteration. Importantly, the first iteration of the Leiden algorithm is the most computationally intensive one, and subsequent iterations are faster. For example, for the Web of Science network, the first iteration takes about 110–120 seconds, while subsequent iterations require about 40 seconds.

## Discussion

Community detection is an important task in the analysis of complex networks. Finding communities in large networks is far from trivial: algorithms need to be fast, but they also need to provide high-quality results. One of the most widely used algorithms is the Louvain algorithm^10^, which is reported to be among the fastest and best performing community detection algorithms^11,12^. However, as shown in this paper, the Louvain algorithm has a major shortcoming: the algorithm yields communities that may be arbitrarily badly connected. Communities may even be disconnected.

To overcome the problem of arbitrarily badly connected communities, we introduced a new algorithm, which we refer to as the Leiden algorithm. This algorithm provides a number of explicit guarantees. In particular, it yields communities that are guaranteed to be connected. Moreover, when the algorithm is applied iteratively, it converges to a partition in which all subsets of all communities are guaranteed to be locally optimally assigned. In practical applications, the Leiden algorithm convincingly outperforms the Louvain algorithm, both in terms of speed and in terms of quality of the results, as shown by the experimental analysis presented in this paper. We conclude that the Leiden algorithm is strongly preferable to the Louvain algorithm.

## Supplementary information

Supplementary Information

LaTeX Supplementary File
