# Supplementary material for: From Louvain to Leiden: guaranteeing well-connected communities
Source: Sci Rep. 2019 Mar 26;9:5233. doi: 10.1038/s41598-019-41695-z (PMC6435756; doi:10.1038/s41598-019-41695-z)
Supplement: Supplementary file 1 — Supplementary Information [file 41598_2019_41695_MOESM1_ESM.pdf]

Supplementary Information
From Louvain to Leiden: guaranteeing well-connected communities

V.A. Traag,* L. Waltman, and N.J. van Eck
Centre for Science and Technology Studies, Leiden University, the Netherlands

Appendix A: Pseudo-code and mathematical notation

Pseudo-code for the Louvain algorithm and the Leiden algorithm is provided in Algorithms A.1 and A.2, respectively. Below we discuss the mathematical notation that is used in the pseudo-code and also in the mathematical results presented in Appendices C, D, and E. There are some uncommon elements in the notation. In particular, the idea of sets of sets plays an important role, and some concepts related to this idea need to be introduced.

Let $G = (V, E)$ be a graph with $n = |V|$ nodes and $m = |E|$ edges. Graphs are assumed to be undirected. With the exception of Theorem 14 in Appendix E, the mathematical results presented in this paper apply to both unweighted and weighted graphs. For simplicity, our mathematical notation assumes graphs to be unweighted, although the notation does allow for multigraphs. A partition $\mathcal{P} = \{C_1, \ldots, C_r\}$ consists of $r = |\mathcal{P}|$ communities, where each community $C_i \subseteq V$ consists of a set of nodes such that $V = \bigcup_i C_i$ and $C_i \cap C_j = \emptyset$ for all $i \neq j$. For two sets R and S, we sometimes use $R + S$ to denote the union $R \cup S$ and $R - S$ to denote the difference $R \setminus S$.

A quality function $\mathcal{H}(G, \mathcal{P})$ assigns a "quality" to a partition $\mathcal{P}$ of a graph G. We aim to find a partition with the highest possible quality. The graph G is often clear from the context, and we therefore usually write $\mathcal{H}(\mathcal{P})$ instead of $\mathcal{H}(G, \mathcal{P})$. Based on partition $\mathcal{P}$, graph G can be *aggregated* into a new graph G'. Graph G is then called the *base graph*, while graph G' is called the *aggregate graph*. The nodes of the aggregate graph G' are the communities in the partition $\mathcal{P}$ of the base graph G, i.e. $V(G') = \mathcal{P}$. The edges of the aggregate graph G' are multi-edges. The number of edges between two nodes in the aggregate graph G' equals the number of edges between nodes in the two corresponding communities in the base graph G. Hence, $E(G') = \{(C, D) \mid (u, v) \in E(G), u \in C \in \mathcal{P}, v \in D \in \mathcal{P}\}$, where $E(G')$ is a multiset. A quality function must have the property that $\mathcal{H}(G, \mathcal{P}) = \mathcal{H}(G', \mathcal{P}')$, where $\mathcal{P}' = \{\{v\} \mid v \in V(G')\}$ denotes the singleton partition of the aggregate graph G'. This ensures that a quality function gives consistent results for base graphs and aggregate graphs.

We denote by $\mathcal{P}(v \mapsto C)$ the partition that is obtained when we start from partition $\mathcal{P}$ and we then move node v to community C. We write $\Delta\mathcal{H}_{\mathcal{P}}(v \mapsto C)$ for the change in the quality function by moving node v to community C for some partition $\mathcal{P}$. In other words, $\Delta\mathcal{H}_{\mathcal{P}}(v \mapsto C) = \mathcal{H}(\mathcal{P}(v \mapsto C)) - \mathcal{H}(\mathcal{P})$. We usually leave the partition $\mathcal{P}$ implicit and simply write $\Delta\mathcal{H}(v \mapsto C)$. Similarly, we denote by $\Delta\mathcal{H}_{\mathcal{P}}(S \mapsto C)$ the change in the quality function by moving a set of nodes S to community C. An empty community is denoted by $\emptyset$. Hence, $\Delta\mathcal{H}_{\mathcal{P}}(S \mapsto \emptyset)$ is the change in the quality function by moving a set of nodes S to an empty (i.e. new) community.

Now consider a community C that consists of two parts S_1 and S_2 such that $C = S_1 \cup S_2$ and $S_1 \cap S_2 = \emptyset$. Suppose that S_1 and S_2 are disconnected. In other words, there are no edges between nodes in S_1 and S_2. We then require a quality function to have the property that $\Delta\mathcal{H}(S_1 \mapsto \emptyset) > 0$ and $\Delta\mathcal{H}(S_2 \mapsto \emptyset) > 0$. This guarantees that a partition can always be improved by splitting a community into its connected components. This comes naturally for most definitions of a community, but this is not the case when considering for example negative links [1].

Because nodes in an aggregate graph are sets themselves, it is convenient to define some recursive properties.

Definition 1. The *recursive size* of a set S is defined as

$$\|S\| = \sum_{s \in S} \|s\|, \tag{A1}$$

where $\|s\| = 1$ if s is not a set itself. The *flattening* operation for a set S is defined as

$$\mathrm{flat}(S) = \bigcup_{s \in S} \mathrm{flat}(s), \tag{A2}$$

where $\mathrm{flat}(s) = s$ if s is not a set itself. A set that has been flattened is called a *flat set*.

* v.a.traag@cwts.leidenuniv.nl

The recursive size of a set corresponds to the usual definition of set size in case the elements of a set are not sets themselves, but it generalizes this definition whenever the elements are sets themselves. For example, if $S = \{\{a, b\}, \{c\}, \{d, e, f\}\}$, then

$$\begin{aligned}\|S\| &= \|\{a,b\}\| + \|\{c\}\| + \|\{d,e,f\}\| \\ &= (\|a\| + \|b\|) + \|c\| + (\|d\| + \|e\| + \|f\|) \\ &= 2 + 1 + 3 = 6.\end{aligned}$$

This contrasts with the traditional size of a set, which is $|S| = 3$, because S contains 3 elements. The fact that the elements are sets themselves plays no role in the traditional size of a set. The flattening of S is

$$\begin{aligned}\text{flat}(S) &= \text{flat}(\{a,b\}) \cup \text{flat}(\{c\}) \cup \text{flat}(\{d,e,f\}) \\ &= a \cup b \cup c \cup d \cup e \cup f \\ &= \{a,b,c,d,e,f\}.\end{aligned}$$

Note that $\|S\| = |\,\text{flat}(S)|$.

Definition 2. The *flattening* operation for a partition $\mathcal{P}$ is defined as

$$\text{flat}^*(\mathcal{P}) = \{\text{flat}(C) \mid C \in \mathcal{P}\}. \tag{A3}$$

Hence, $\text{flat}^*(\mathcal{P})$ denotes the operation in which each community $C \in \mathcal{P}$ is flattened. A partition that has been flattened is called a *flat partition*.

For any partition of an aggregate graph, the equivalent partition of the base graph can be obtained by applying the flattening operation.

Additionally, we need some terminology to describe the connectivity of communities.

Definition 3. Let $G = (V, E)$ be a graph, and let $\mathcal{P}$ be a partition of G. Furthermore, let $H(C)$ be the subgraph induced by a community $C \in \mathcal{P}$, i.e. $V(H) = C$ and $E(H) = \{(u, v) \mid (u, v) \in E(G), u, v \in C\}$. A community $C \in \mathcal{P}$ is called *connected* if $H(C)$ is a connected graph. Conversely, a community $C \in \mathcal{P}$ is called *disconnected* if $H(C)$ is a disconnected graph.

The mathematical proofs presented in this paper rely on the Constant Potts Model (CPM) [2]. This quality function has important advantages over modularity. In particular, unlike modularity, CPM does not suffer from the problem of the resolution limit [2, 3]. Moreover, our mathematical definitions and proofs are quite elegant when expressed in terms of CPM. The CPM quality function is defined as

$$\mathcal{H}(G, \mathcal{P}) = \sum_{C \in \mathcal{P}} \left[E(C, C) - \gamma \binom{\|C\|}{2} \right], \tag{A4}$$

where $E(C, D) = |\{(u, v) \in E(G) \mid u \in C, v \in D\}|$ denotes the number of edges between nodes in communities C and D. Note that this definition can also be used for aggregate graphs because $E(G)$ is a multiset.

The mathematical results presented in this paper also extend to modularity, although the formulations are less elegant. Results for modularity are straightforward to prove by redefining the recursive size $\|S\|$ of a set S. We need to define the size of a node v in the base graph as $\|v\| = k_v$ instead of $\|v\| = 1$, where k_v is the degree of node v. Furthermore, we need to rescale the resolution parameter γ by $2m$. Modularity can then be written as

$$\mathcal{H}(G, \mathcal{P}) = \sum_{C \in \mathcal{P}} \left[E(C, C) - \frac{\gamma}{2m} \binom{\|C\|}{2} \right]. \tag{A5}$$

Note that, in addition to the overall multiplicative factor of $\frac{1}{2m}$, this adds a constant $\frac{\gamma}{2m} \sum_C \frac{\|C\|}{2} = \frac{\gamma}{2}$ to the ordinary definition of modularity [4]. However, this does not matter for optimisation or for the proofs.

As discussed in the main text, the Louvain and the Leiden algorithm can be *iterated* by performing multiple consecutive iterations of the algorithm, using the partition identified in one iteration as starting point for the next iteration. In this way, a sequence of partitions $\mathcal{P}_0, \mathcal{P}_1, \ldots$ is obtained such that $\mathcal{P}_{t+1} = \textsc{Louvain}(G, \mathcal{P}_t)$ or $\mathcal{P}_{t+1} = \textsc{Leiden}(G, \mathcal{P}_t)$. The initial partition $\mathcal{P}_0$ usually is the singleton partition of the graph G, i.e. $\mathcal{P}_0 = \{\{v\} \mid v \in V\}$.

```
function Louvain(Graph G, Partition 𝒫)
    do
        𝒫 ← MoveNodes(G, 𝒫)                    ▷ Move nodes between communities
        done ← |𝒫| = |V(G)|                     ▷ Terminate when each community consists of only one node
        if not done then
            G ← AggregateGraph(G, 𝒫)            ▷ Create aggregate graph based on partition 𝒫
            𝒫 ← SingletonPartition(G)           ▷ Assign each node in aggregate graph to its own community
        end if
    while not done
    return flat*(𝒫)
end function

function MoveNodes(Graph G, Partition 𝒫)
    do
        ℋ_old = ℋ(𝒫)
        for v ∈ V(G) do                         ▷ Visit nodes (in random order)
            C′ ← arg max_{C∈𝒫∪∅} Δℋ_𝒫(v ↦ C)    ▷ Determine best community for node v
            if Δℋ_𝒫(v ↦ C′) > 0 then            ▷ Perform only strictly positive node movements
                v ↦ C′                          ▷ Move node v to community C′
            end if
        end for
    while ℋ(𝒫) > ℋ_old                          ▷ Continue until no more nodes can be moved
    return 𝒫
end function

function AggregateGraph(Graph G, Partition 𝒫)
    V ← 𝒫                                       ▷ Communities become nodes in aggregate graph
    E ← {(C, D) | (u, v) ∈ E(G), u ∈ C ∈ 𝒫, v ∈ D ∈ 𝒫}   ▷ E is a multiset
    return Graph(V, E)
end function

function SingletonPartition(Graph G)
    return {{v} | v ∈ V(G)}                     ▷ Assign each node to its own community
end function
```

ALGORITHM A.1. **Louvain algorithm.**

Appendix B: Disconnected communities in the Louvain algorithm

In this appendix, we analyse the problem that communities obtained using the Louvain algorithm may be disconnected. This problem is also discussed in the main text, using the example presented in Fig. 2. However, the main text offers no numerical details. These details are provided below.

We consider the CPM quality function with a resolution of $\gamma = \frac{1}{7}$. In the example presented in Fig. 2, the edges between nodes 0 and 1 and between nodes 0 and 4 have a weight of 2, as indicated by the thick lines in the figure. All other edges have a weight of 1. The Louvain algorithm starts from a singleton partition, with each node being assigned to its own community. The algorithm then keeps iterating over all nodes, moving each node to its optimal community. Depending on the order in which the nodes are visited, the following could happen. Node 1 is visited first, followed by node 4. Nodes 1 and 4 join the community of node 0, because the weight of the edges between nodes 0 and 1 and between nodes 0 and 4 is sufficiently high. For node 1, the best move clearly is to join the community of node 0. For node 4, the benefit of joining the community of nodes 0 and 1 then is $2-\gamma\cdot 2 = \frac{12}{7}$. This is larger than the benefit of joining the community of node 5 or 6, which is $1-\gamma\cdot 1 = \frac{6}{7}$. Next, nodes 2, 3, 5 and 6 are visited. For these nodes, it is beneficial to join the community of nodes 0, 1 and 4, because joining this community has a benefit of at least $1-\gamma\cdot 6 = \frac{1}{7} > 0$. This then yields the situation portrayed in Fig. 2(a). After some node movements in the rest of the graph, some neighbours of node 0 in the rest of the graph end up together in a new community. Consequently, when node 0 is visited, it can best be moved to this new community, which gives the situation depicted in Fig. 2(b). In particular, suppose there are 5 nodes in the new community, all of which are connected to node 0. In that case, the benefit for node 0 of moving to this community is $5-\gamma\cdot 5 = \frac{30}{7}$, while the benefit of staying in the current community is only $2\cdot 2-\gamma\cdot 6 = \frac{22}{7}$. After node 0 has moved, nodes 1 and 4 are still locally optimally assigned. For these nodes, the benefit of moving to the new community of node 0 is $2-\gamma\cdot 6 = \frac{8}{7}$. This is smaller than the benefit of staying in the current community, which is $2-\gamma\cdot 5 = \frac{9}{7}$. Finally, nodes 2, 3, 5 and 6 are all locally optimally assigned, as

function LEIDEN(Graph G, Partition $\mathcal{P}$)
    do
        $\mathcal{P} \leftarrow$ MOVENODESFAST$(G, \mathcal{P})$ ▷ Move nodes between communities
        done $\leftarrow |\mathcal{P}| = |V(G)|$ ▷ Terminate when each community consists of only one node
        if not done **then**
            $\mathcal{P}_{\text{refined}} \leftarrow$ REFINEPARTITION$(G, \mathcal{P})$ ▷ Refine partition $\mathcal{P}$
            $G \leftarrow$ AGGREGATEGRAPH$(G, \mathcal{P}_{\text{refined}})$ ▷ Create aggregate graph based on refined partition $\mathcal{P}_{\text{refined}}$
            $\mathcal{P} \leftarrow \{\{v \mid v \subseteq C, v \in V(G)\} \mid C \in \mathcal{P}\}$ ▷ But maintain partition $\mathcal{P}$
        end if
    while not done
    return flat$^*(\mathcal{P})$
end function

function MOVENODESFAST(Graph G, Partition $\mathcal{P}$)
    $Q \leftarrow$ QUEUE$(V(G))$ ▷ Make sure that all nodes will be visited (in random order)
    do
        $v \leftarrow Q$.remove() ▷ Determine next node to visit
        $C' \leftarrow \arg\max_{C \in \mathcal{P} \cup \emptyset} \Delta\mathcal{H}_{\mathcal{P}}(v \mapsto C)$ ▷ Determine best community for node v
        if $\Delta\mathcal{H}_{\mathcal{P}}(v \mapsto C') > 0$ **then** ▷ Perform only strictly positive node movements
            $v \mapsto C'$ ▷ Move node v to community C'
            $N \leftarrow \{u \mid (u, v) \in E(G), u \notin C'\}$ ▷ Identify neighbours of node v that are not in community C'
            Q.add$(N - Q)$ ▷ Make sure that these neighbours will be visited
        end if
    while $Q \neq \emptyset$ ▷ Continue until there are no more nodes to visit
    return $\mathcal{P}$
end function

function REFINEPARTITION(Graph G, Partition $\mathcal{P}$)
    $\mathcal{P}_{\text{refined}} \leftarrow$ SINGLETONPARTITION(G) ▷ Assign each node to its own community
    for $C \in \mathcal{P}$ **do** ▷ Visit communities
        $\mathcal{P}_{\text{refined}} \leftarrow$ MERGENODESSUBSET$(G, \mathcal{P}_{\text{refined}}, C)$ ▷ Refine community C
    end for
    return $\mathcal{P}_{\text{refined}}$
end function

function MERGENODESSUBSET(Graph G, Partition $\mathcal{P}$, Subset S)
    $R = \{v \mid v \in S, E(v, S - v) \geq \gamma \|v\| \cdot (\|S\| - \|v\|)\}$ ▷ Consider only nodes that are well connected within subset S
    for $v \in R$ **do** ▷ Visit nodes (in random order)
        if v in singleton community **then** ▷ Consider only nodes that have not yet been merged
            $\mathcal{T} \leftarrow \{C \mid C \in \mathcal{P}, C \subseteq S, E(C, S - C) \geq \gamma \|C\| \cdot (\|S\| - \|C\|)\}$ ▷ Consider only well-connected communities
            $\Pr(C' = C) \sim \begin{cases} \exp\left(\frac{1}{\theta}\Delta\mathcal{H}_{\mathcal{P}}(v \mapsto C)\right) & \text{if } \Delta\mathcal{H}_{\mathcal{P}}(v \mapsto C) \geq 0 \\ 0 & \text{otherwise} \end{cases} \quad \text{for } C \in \mathcal{T}$ ▷ Choose random community C'
            $v \mapsto C'$ ▷ Move node v to community C'
        end if
    end for
    return $\mathcal{P}$
end function

function AGGREGATEGRAPH(Graph G, Partition $\mathcal{P}$)
    $V \leftarrow \mathcal{P}$ ▷ Communities become nodes in aggregate graph
    $E \leftarrow \{(C, D) \mid (u, v) \in E(G), u \in C \in \mathcal{P}, v \in D \in \mathcal{P}\}$ ▷ E is a multiset
    return GRAPH(V, E)
end function

function SINGLETONPARTITION(Graph G)
    return $\{\{v\} \mid v \in V(G)\}$ ▷ Assign each node to its own community
end function

ALGORITHM A.2. **Leiden algorithm.**

$1 - \gamma \cdot 5 = \frac{2}{7} > 0$. Hence, we end up with a community that is disconnected. In later stages of the Louvain algorithm, there will be no possibility to repair this.

The example presented above considers a weighted graph, but this graph can be assumed to be an aggregate graph of an unweighted base graph, thus extending the example also to unweighted graphs. Although the example uses the CPM quality function, similar examples can be given for modularity. However, because of the dependency of modularity on the number of edges m, the calculations for modularity are a bit more complex. Importantly, both for CPM and for modularity, the Louvain algorithm suffers from the problem of disconnected communities.

Appendix C: Reachability of optimal partitions

In this appendix, we consider two types of move sequences: non-decreasing move sequences and greedy move sequences. For each type of move sequence, we study whether all optimal partitions are reachable. We first show that this is not the case for greedy move sequences. In particular, we show that for some optimal partitions there does not exist a greedy move sequence that is able to reach the partition. We then show that optimal partitions can always be reached using a non-decreasing move sequence. This result forms the basis for the asymptotic guarantees of the Leiden algorithm, which are discussed in Appendix D 3.

We first define the different types of move sequences.

Definition 4. Let $G = (V, E)$ be a graph, and let $\mathcal{P}_0, \ldots, \mathcal{P}_\tau$ be partitions of G. A sequence of partitions $\mathcal{P}_0, \ldots, \mathcal{P}_\tau$ is called a *move sequence* if for each $t = 0, \ldots, \tau - 1$ there exists a node $v_t \in V$ and a community $C_t \in \mathcal{P}_t \cup \emptyset$ such that $\mathcal{P}_{t+1} = \mathcal{P}_t(v_t \mapsto C_t)$. A move sequence is called *non-decreasing* if $\mathcal{H}(\mathcal{P}_{t+1}) \geq \mathcal{H}(\mathcal{P}_t)$ for all $t = 0, \ldots, \tau - 1$. A move sequence is called *greedy* if $\mathcal{H}(\mathcal{P}_{t+1}) = \max_C \mathcal{H}(\mathcal{P}_t(v_t \mapsto C))$ for all $t = 0, \ldots, \tau - 1$.

In other words, the next partition in a move sequence is obtained by moving a single node to a different community. Clearly, a greedy move sequence must be non-decreasing, but a non-decreasing move sequence does not need to be greedy. A natural question is whether for any optimal partition $\mathcal{P}^*$ there exists a move sequence that starts from the singleton partition and that reaches the optimal partition, i.e., a move sequence $\mathcal{P}_0, \ldots, \mathcal{P}_\tau$ with $\mathcal{P}_0 = \{\{v\} \mid v \in V\}$ and $\mathcal{P}_\tau = \mathcal{P}^*$. Trivially, it is always possible to reach the optimal partition if we allow all moves—even moves that decrease the quality function—as is done for example in simulated annealing [5, 6]. However, it can be shown that there is no need to consider all moves in order to reach the optimal partition. It is sufficient to consider only non-decreasing moves. On the other hand, considering only greedy moves turns out to be too restrictive to guarantee that the optimal partition can be reached.

1. Non-decreasing move sequences

We here prove that for any graph there exists a non-decreasing move sequence that reaches the optimal partition $\mathcal{P}^*$. The optimal partition can be reached in $n - |\mathcal{P}^*|$ steps.

Theorem 1. Let $G = (V, E)$ be a graph, and let $\mathcal{P}^*$ be an optimal partition of G. There then exists a non-decreasing move sequence $\mathcal{P}_0, \ldots, \mathcal{P}_\tau$ with $\mathcal{P}_0 = \{\{v\} \mid v \in V\}$, $\mathcal{P}_\tau = \mathcal{P}^*$, and $\tau = n - |\mathcal{P}^*|$.

Proof. Let $C^* \in \mathcal{P}^*$ be a community in the optimal partition $\mathcal{P}^*$, let $v_0 \in C^*$ be a node in this community, and let $C_0 = \{v_0\}$. Let $\mathcal{P}_0 = \{\{v\} \mid v \in V\}$ be the singleton partition. For $t = 1, \ldots, |C^*| - 1$, let $v_t \in C^* - C_{t-1}$, let $C_t = \{v_0, \ldots, v_t\} \in \mathcal{P}_t$, and let $\mathcal{P}_t = \mathcal{P}_{t-1}(v_t \mapsto C_{t-1})$. We prove by contradiction that there always exists a non-decreasing move sequence $\mathcal{P}_0, \ldots, \mathcal{P}_{|C^*|-1}$. Assume that for some t there does not exist a node v_t for which $\Delta\mathcal{H}(v_t \mapsto C_{t-1}) \geq 0$. Let $S = C^* - C_{t-1}$ and $R = C_{t-1}$. For all $v \in S$,

$$E(v, R) - \gamma\|v\| \cdot \|R\| < 0.$$

This implies that

$$E(S, R) = \sum_{v \in S} E(v, R) < \gamma\|S\| \cdot \|R\|.$$

However, by optimality, for all $S \subseteq C^*$ and $R = C^* - S$,

$$E(S, R) \geq \gamma\|S\| \cdot \|R\|.$$

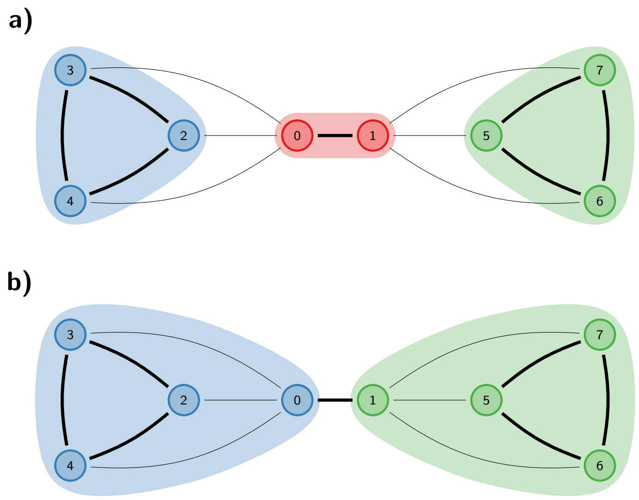

FIG. C.1. **Unreachable optimal partition.** A greedy move sequence always reaches the partition in (a), whereas the partition in (b) is optimal. This demonstrates that for some graphs there does not exist a greedy move sequence that reaches the optimal partition.

We therefore have a contradiction. Hence, there always exists a non-decreasing move sequence $\mathcal{P}_0, \ldots, \mathcal{P}_{|C^*|-1}$. This move sequence reaches the community $C_t = C^*$. The above reasoning can be applied to each community $C^* \in \mathcal{P}^*$. Consequently, each of these communities can be reached using a non-decreasing move sequence. In addition, for each community $C^* \in \mathcal{P}^*$, this can be done in $|C^*| - 1$ steps, so that in total $\tau = \sum_{C^* \in \mathcal{P}^*}(|C^*| - 1) = n - |\mathcal{P}^*|$ steps are needed. ■

2. Greedy move sequences

We here show that there does not always exist a greedy move sequence that reaches the optimal partition of a graph. To show this, we provide a counterexample in which we have a graph for which there is no greedy move sequence that reaches the optimal partition. Our counterexample includes two nodes that should be assigned to different communities. However, because there is a strong connection between the nodes, in a greedy move sequence the nodes are always assigned to the same community. We use the CPM quality function in our counterexample, but a similar counterexample can be given for modularity. The counterexample is illustrated in Fig. C.1. The thick edges have a weight of 3, while the thin ones have a weight of $\frac{3}{2}$. The resolution is set to $\gamma = 1$. In this situation, nodes 0 and 1 are always joined together in a community. This has a benefit of $3 - \gamma = 2$, which is larger than the benefit of $3 \cdot \frac{3}{2} - \gamma \cdot 3 = \frac{3}{2}$ obtained by node 0 joining the community of nodes 2, 3 and 4 or node 1 joining the community of nodes 5, 6 and 7. Hence, regardless of the exact node order, the partition reached by a greedy move sequence always consists of three communities. This gives a total quality of

$$2 \cdot \left(3 \cdot 3 - \gamma \frac{3 \cdot 2}{2}\right) + \left(3 - \gamma \frac{2 \cdot 1}{2}\right) = 14,$$

while the optimal partition has only two communities, consisting of nodes $\{0, 2, 3, 4\}$ and $\{1, 5, 6, 7\}$ and resulting in a total quality of

$$2 \cdot \left(3 \cdot 3 + 3 \cdot \frac{3}{2} - \gamma \frac{4 \cdot 3}{2}\right) = 15.$$

Hence, a greedy move sequence always reaches the partition in Fig. C.1(a), whereas the partition in Fig. C.1(b) is optimal.

Appendix D: Guarantees of the Leiden algorithm

In this appendix, we discuss the guarantees provided by the Leiden algorithm. The guarantees of the Leiden algorithm partly rely on the randomness in the algorithm. We therefore require that $\theta > 0$. Before stating the guarantees of the Leiden algorithm, we first define a number of properties. We start by introducing some relatively weak properties, and we then move on to stronger properties. In the following definitions, $\mathcal{P}$ is a flat partition of a graph $G = (V, E)$.

Definition 5 (γ-separation)**.** We call a pair of communities $C, D \in \mathcal{P}$ *γ-separated* if $\Delta\mathcal{H}(C \mapsto D) = \Delta\mathcal{H}(D \mapsto C) \leq 0$. A community $C \in \mathcal{P}$ is γ-separated if C is γ-separated with respect to all $D \in \mathcal{P}$. A partition $\mathcal{P}$ is γ-separated if all $C \in \mathcal{P}$ are γ-separated.

Definition 6 (γ-connectivity)**.** We call a set of nodes $S \subseteq C \in \mathcal{P}$ *γ-connected* if $|S| = 1$ or if S can be partitioned into two sets R and T such that $E(R, T) \geq \gamma\|R\| \cdot \|T\|$ and R and T are γ-connected. A community $C \in \mathcal{P}$ is γ-connected if $S = C$ is γ-connected. A partition $\mathcal{P}$ is γ-connected if all $C \in \mathcal{P}$ are γ-connected.

Definition 7 (Subpartition γ-density)**.** We call a set of nodes $S \subseteq C \in \mathcal{P}$ *subpartition γ-dense* if the following two conditions are satisfied: (i) $\Delta\mathcal{H}(S \mapsto \emptyset) \leq 0$ and (ii) $|S| = 1$ or S can be partitioned into two sets R and T such that $E(R, T) \geq \gamma\|R\| \cdot \|T\|$ and R and T are subpartition γ-dense. A community $C \in \mathcal{P}$ is subpartition γ-dense if $S = C$ is subpartition γ-dense. A partition $\mathcal{P}$ is subpartition γ-dense if all $C \in \mathcal{P}$ are subpartition γ-dense.

Definition 8 (Node optimality)**.** We call a community $C \in \mathcal{P}$ *node optimal* if $\Delta\mathcal{H}(v \mapsto D) \leq 0$ for all $v \in C$ and all $D \in \mathcal{P}$ (or $D = \emptyset$). A partition $\mathcal{P}$ is node optimal if all $C \in \mathcal{P}$ are node optimal.

Definition 9 (Uniform γ-density)**.** We call a community $C \in \mathcal{P}$ *uniformly γ-dense* if $\Delta\mathcal{H}(S \mapsto \emptyset) \leq 0$ for all $S \subseteq C$. A partition $\mathcal{P}$ is uniformly γ-dense if all $C \in \mathcal{P}$ are uniformly γ-dense.

Definition 10 (Subset optimality)**.** We call a community $C \in \mathcal{P}$ *subset optimal* if $\Delta\mathcal{H}(S \mapsto D) \leq 0$ for all $S \subseteq C$ and all $D \in \mathcal{P}$ (or $D = \emptyset$). A partition $\mathcal{P}$ is subset optimal if all $C \in \mathcal{P}$ are subset optimal.

Subset optimality clearly is the strongest property and subsumes all other properties. Uniform γ-density is subsumed by subset optimality but may be somewhat more intuitive to grasp. It states that any subset of nodes in a community is always connected to the rest of the community with a density of at least γ. In other words, for all $S \subseteq C \in \mathcal{P}$ we have

$$E(S, C - S) \geq \gamma\|S\| \cdot \|C - S\|. \tag{D1}$$

Imposing the restriction $D = \emptyset$ in the definition of subset optimality gives the property of uniform γ-density, restricting S to consist of only one node gives the property of node optimality, and imposing the restriction $S = C$ yields the property of γ-separation. Uniform γ-density implies subpartition γ-density, which in turn implies γ-connectivity. Subpartition γ-density also implies that individual nodes cannot be split from their community (but notice that this is a weaker property than node optimality). Ordinary connectivity is implied by γ-connectivity, but not vice versa. Obviously, any optimal partition is subset optimal, but not the other way around: a subset optimal partition is not necessarily an optimal partition (see Fig. C.1(a) for an example).

In the rest of this appendix, we show that the Leiden algorithm guarantees that the above properties hold for partitions produced by the algorithm. The properties hold either in each iteration, in every stable iteration, or asymptotically. The first two properties of γ-separation and γ-connectivity are guaranteed in each iteration of the Leiden algorithm. We prove this in Appendix D 1. The next two properties of subpartition γ-density and node optimality are guaranteed in every stable iteration of the Leiden algorithm, as we prove in Appendix D 2. Finally, in Appendix D 3 we prove that asymptotically the Leiden algorithm guarantees the last two properties of uniform γ-density and subset optimality.

1. Guarantees in each iteration

In order to show that the property of γ-separation is guaranteed in each iteration of the Leiden algorithm, we first need to prove some results for the MoveNodesFast function in the Leiden algorithm.

We start by introducing some notation. The MoveNodesFast function iteratively evaluates nodes. When a node is evaluated, either it is moved to a different (possibly empty) community or it is kept in its current community, depending on what is most beneficial for the quality function. Let $G = (V, E)$ be a graph, let $\mathcal{P}$ be a partition of G, and let $\mathcal{P}' = \text{MoveNodesFast}(G, \mathcal{P})$. We denote by $\mathcal{P}_0, \ldots, \mathcal{P}_r$ a sequence of partitions generated by the

MoveNodesFast function, with $\mathcal{P}_0 = \mathcal{P}$ denoting the initial partition, $\mathcal{P}_1$ denoting the partition after the first evaluation of a node has taken place, and so on. $\mathcal{P}_r = \mathcal{P}'$ denotes the partition after the final evaluation of a node has taken place. The MoveNodesFast function maintains a queue of nodes that still need to be evaluated. Let Q_s be the set of nodes that still need to be evaluated after s node evaluations have taken place, with $Q_0 = V$. Also, for all $v \in V$, let $C_s^v \in \mathcal{P}_s$ be the community in which node v finds itself after s node evaluations have taken place.

The following lemma states that at any point in the MoveNodesFast function, if a node is disconnected from the rest of its community, the node will find itself in the queue of nodes that still need to be evaluated.

Lemma 2. Using the notation introduced above, for all $v \in V$ and all s, we have $v \in Q_s$ or $|C_s^v| = 1$ or $E(v, C_s^v - v) > 0$.

Proof. We are going to prove the lemma for an arbitrary node $v \in V$. We provide a proof by induction. We observe that $v \in Q_0$, which provides our inductive base. Suppose that $v \in Q_{s-1}$ or $|C_{s-1}^v| = 1$ or $E(v, C_{s-1}^v - v) > 0$. This is our inductive hypothesis. We are going to show that $v \in Q_s$ or $|C_s^v| = 1$ or $E(v, C_s^v - v) > 0$. If $v \in Q_s$, this result is obtained in a trivial way. Suppose therefore that $v \notin Q_s$. We then need to show that $|C_s^v| = 1$ or $E(v, C_s^v - v) > 0$. To do so, we distinguish between two cases.

We first consider the case in which $v \in Q_{s-1}$. If $v \in Q_{s-1}$ and $v \notin Q_s$, node v has just been evaluated. We then obviously have $|C_s^v| = 1$ or $E(v, C_s^v - v) > 0$. Otherwise we would have $|C_s^v| > 1$ and $E(v, C_s^v - v) = 0$, which would mean that node v is disconnected from the rest of its community. Since node v has just been evaluated, this is not possible.

We now consider the case in which $v \notin Q_{s-1}$. Let $u \in V$ be the node that has just been evaluated, i.e., $u \in Q_{s-1}$ and $u \notin Q_s$. If node u has not been moved to a different community, then $\mathcal{P}_s = \mathcal{P}_{s-1}$. Obviously, if $|C_{s-1}^v| = 1$ or $E(v, C_{s-1}^v - v) > 0$, we then have $|C_s^v| = 1$ or $E(v, C_s^v - v) > 0$. On the other hand, if node u has been moved to a different community, we have $(u, v) \notin E(G)$ or $v \in C_s^u$. To see this, note that if $(u, v) \in E(G)$ and $v \notin C_s^u$, we would have $v \in Q_s$ (following line 21 in Algorithm A.2). This contradicts our assumption that $v \notin Q_s$, so that we must have $(u, v) \notin E(G)$ or $v \in C_s^u$. In other words, either there is no edge between nodes u and v or node u has been moved to the community of node v. In either case, it is not possible that the movement of node u causes node v to become disconnected from the rest of its community. Hence, in either case, if $|C_{s-1}^v| = 1$ or $E(v, C_{s-1}^v - v) > 0$, then $|C_s^v| = 1$ or $E(v, C_s^v - v) > 0$. ■

Using Lemma 2, we now prove the following lemma, which states that for partitions provided by the MoveNodesFast function it is guaranteed that singleton communities cannot be merged with each other.

Lemma 3. Let $G = (V, E)$ be a graph, let $\mathcal{P}$ be a partition of G, and let $\mathcal{P}' = \text{MoveNodesFast}(G, \mathcal{P})$. Then for all pairs $C, D \in \mathcal{P}'$ such that $|C| = |D| = 1$, we have $\Delta\mathcal{H}(C \mapsto D) = \Delta\mathcal{H}(D \mapsto C) \leq 0$.

Proof. We are going to prove the lemma for an arbitrary pair of communities $C, D \in \mathcal{P}'$ such that $|C| = |D| = 1$. We use the notation introduced above. If $C, D \in \mathcal{P}_s$ for all s, it is clear that $\Delta\mathcal{H}(C \mapsto D) = \Delta\mathcal{H}(D \mapsto C) \leq 0$. Otherwise, consider t such that $C, D \in \mathcal{P}_s$ for all $s \geq t$ and either $C \notin \mathcal{P}_{t-1}$ or $D \notin \mathcal{P}_{t-1}$. Without loss of generality, we assume that $C \notin \mathcal{P}_{t-1}$ and $D \in \mathcal{P}_{t-1}$. Consider $v \in V$ such that $C = \{v\}$. After $t-1$ node evaluations have taken place, there are two possibilities.

One possibility is that node v is evaluated and is moved to an empty community. This means that moving node v to an empty community is more beneficial for the quality function than moving node v to community D. It is then clear that $\Delta\mathcal{H}(C \mapsto D) = \Delta\mathcal{H}(D \mapsto C) \leq 0$.

The second possibility is that node v is in a community together with one other node $u \in V$ (i.e. $\{u, v\} \in \mathcal{P}_{t-1}$) and that this node u is evaluated and is moved to a different community. In this case, $v \in Q_t$, as we will now show. If $(u, v) \in E(G)$, this follows from line 21 in Algorithm A.2. If $(u, v) \notin E(G)$, we have $|C_{t-1}^v| = |\{u, v\}| = 2$ and $E(v, C_{t-1}^v - v) = 0$. It then follows from Lemma 2 that $v \in Q_{t-1}$. Since node v is not evaluated in node evaluation t (node u is evaluated in this node evaluation), $v \in Q_{t-1}$ implies that $v \in Q_t$. If $v \in Q_t$, at some point $s \geq t$, node v is evaluated. Since $C, D \in \mathcal{P}_s$ for all $s \geq t$, keeping node v in its own singleton community C is more beneficial for the quality function than moving node v to community D. This means that $\Delta\mathcal{H}(C \mapsto D) = \Delta\mathcal{H}(D \mapsto C) \leq 0$. ■

Lemma 3 enables us to prove that the property of γ-separation is guaranteed in each iteration of the Leiden algorithm, as stated in the following theorem.

Theorem 4. Let $G = (V, E)$ be a graph, let $\mathcal{P}_t$ be a flat partition of G, and let $\mathcal{P}_{t+1} = \text{Leiden}(G, \mathcal{P}_t)$. Then $\mathcal{P}_{t+1}$ is γ-separated.

Proof. Let $G_\ell = (V_\ell, E_\ell)$ be the aggregate graph at the highest level in the Leiden algorithm, let $\mathcal{P}_\ell$ be the initial partition of G_ℓ, and let $\mathcal{P}'_\ell = \text{MoveNodesFast}(G_\ell, \mathcal{P}_\ell)$. Since we are at the highest level of aggregation, it follows from line 4 in Algorithm A.2 that $|\mathcal{P}'_\ell| = |V_\ell|$, which means that $|C| = 1$ for all $C \in \mathcal{P}'_\ell$. In other words, $\mathcal{P}'_\ell$ is a

singleton partition of G_ℓ. Lemma 3 then implies that for all $C, D \in \mathcal{P}'_\ell$ we have $\Delta\mathcal{H}(C \mapsto D) = \Delta\mathcal{H}(D \mapsto C) \leq 0$. Since $\mathcal{P}_{t+1} = \text{flat}^*(\mathcal{P}'_\ell)$, it follows that for all $C, D \in \mathcal{P}_{t+1}$ we have $\Delta\mathcal{H}(C \mapsto D) = \Delta\mathcal{H}(D \mapsto C) \leq 0$. Hence, $\mathcal{P}_{t+1}$ is γ-separated. ■

The property of γ-separation also holds after each iteration of the Louvain algorithm. In fact, for the Louvain algorithm this is much easier to see than for the Leiden algorithm. The Louvain algorithm uses the MoveNodes function instead of the MoveNodesFast function. Unlike the MoveNodesFast function, the MoveNodes function yields partitions that are guaranteed to be node optimal. This guarantee leads in a straightforward way to the property of γ-separation for partitions obtained in each iteration of the Louvain algorithm.

We now consider the property of γ-connectivity. By constructing a tree corresponding to the decomposition of γ-connectivity, we are going to prove that this property is guaranteed in each iteration of the Leiden algorithm.

Theorem 5. Let $G = (V, E)$ be a graph, let $\mathcal{P}_t$ be a flat partition of G, and let $\mathcal{P}_{t+1} = \text{Leiden}(G, \mathcal{P}_t)$. Then $\mathcal{P}_{t+1}$ is γ-connected.

Proof. Let $G_\ell = (V_\ell, E_\ell)$ be the aggregate graph at level ℓ in the Leiden algorithm, with $G_0 = G$ being the base graph. We say that a node $v \in V_\ell$ is γ-connected if $\text{flat}(v)$ is γ-connected. We are going to proceed inductively. Each node in the base graph G_0 is trivially γ-connected. This provides our inductive base. Suppose that each node $v \in V_{\ell-1}$ is γ-connected, which is our inductive hypothesis. Each node $v \in V_\ell$ is obtained by merging one or more nodes at the preceding level, i.e. $v = \{u \mid u \in S\}$ for some set $S \subseteq V_{\ell-1}$. If v consists of only one node at the preceding level, v is immediately γ-connected by our inductive hypothesis. The set of nodes S is constructed in the MergeNodesSubset function. There exists some order $u_1, \ldots, u_k$ in which nodes are added to S. Let $S_i = \{u_1, \ldots, u_i\}$ be the set obtained after adding node u_i. It follows from line 38 in Algorithm A.2 that $E(u_{i+1}, S_i) \geq \gamma \|u_{i+1}\| \cdot \|S_i\|$ for $i = 1, \ldots, k-1$. Taking into account that each u_i is γ-connected by our inductive hypothesis, this implies that each set S_i is γ-connected. Since $S = S_k$ is γ-connected, node v is γ-connected. Hence, each node $v \in V_\ell$ is γ-connected. This also holds for the nodes in the aggregate graph at the highest level in the Leiden algorithm, which implies that all communities in $\mathcal{P}_{t+1}$ are γ-connected. In other words, $\mathcal{P}_{t+1}$ is γ-connected. ■

Note that the theorem does not require $\mathcal{P}_t$ to be connected. Even if a disconnected partition is provided as input to the Leiden algorithm, performing a single iteration of the algorithm will give a partition that is γ-connected.

2. Guarantees in stable iterations

As discussed earlier, the Leiden algorithm can be iterated until $\mathcal{P}_{t+1} = \text{Leiden}(G, \mathcal{P}_t)$. Likewise, the Louvain algorithm can be iterated until $\mathcal{P}_{t+1} = \text{Louvain}(G, \mathcal{P}_t)$. We say that an iteration is *stable* if $\mathcal{P}_{t+1} = \mathcal{P}_t$, in which case we call $\mathcal{P}_t$ (or $\mathcal{P}_{t+1}$) a *stable partition.*

There is a subtle point when considering stable iterations. In order for the below guarantees to hold, we need to ensure that $\mathcal{H}(\mathcal{P}_{t+1}) = \mathcal{H}(\mathcal{P}_t)$ implies $\mathcal{P}_{t+1} = \mathcal{P}_t$. In both the Leiden algorithm and the Louvain algorithm, we therefore consider only strictly positive improvements (see line 17 in Algorithm A.1 and line 18 in Algorithm A.2). In other words, if a node movement leads to a partition that has the same quality as the current partition, the current partition is preferred and the node movement will not take place. This then also implies that $\mathcal{H}(\mathcal{P}_{t+1}) > \mathcal{H}(\mathcal{P}_t)$ if $\mathcal{P}_{t+1} \neq \mathcal{P}_t$.

The Leiden algorithm guarantees that a stable partition is subpartition γ-dense, as stated in the following theorem. Note that the proof of the theorem has a structure that is similar to the structure of the proof of Theorem 5 presented above.

Theorem 6. Let $G = (V, E)$ be a graph, let $\mathcal{P}_t$ be a flat partition of G, and let $\mathcal{P}_{t+1} = \text{Leiden}(G, \mathcal{P}_t)$. If $\mathcal{P}_{t+1} = \mathcal{P}_t$, then $\mathcal{P}_{t+1} = \mathcal{P}_t$ is subpartition γ-dense.

Proof. Suppose we have a stable iteration. Hence, $\mathcal{P}_{t+1} = \mathcal{P}_t$. Let $G_\ell = (V_\ell, E_\ell)$ be the aggregate graph at level ℓ in the Leiden algorithm, with $G_0 = G$ being the base graph. We say that a node $v \in V_\ell$ is subpartition γ-dense if the set of nodes $\text{flat}(v)$ is subpartition γ-dense. We first observe that for all levels ℓ and all nodes $v \in V_\ell$ we have $\Delta\mathcal{H}(v \mapsto \emptyset) \leq 0$. To see this, note that if $\Delta\mathcal{H}(v \mapsto \emptyset) > 0$ for some level ℓ and some node $v \in V_\ell$, the MoveNodesFast function would have removed node v from its community, which means that the iteration would not have been stable. We are now going to proceed inductively. Since $\Delta\mathcal{H}(v \mapsto \emptyset) \leq 0$ for all nodes $v \in V_0$, each node in the base graph G_0 is subpartition γ-dense. This provides our inductive base. Suppose that each node $v \in V_{\ell-1}$ is subpartition γ-dense, which is our inductive hypothesis. Each node $v \in V_\ell$ is obtained by merging one or more nodes at the preceding level, i.e. $v = \{u \mid u \in S\}$ for some set $S \subseteq V_{\ell-1}$. If v consists of only one node at the preceding level, v is immediately subpartition γ-dense by our inductive hypothesis. The set of nodes S is

constructed in the MergeNodesSubset function. There exists some order $u_1, \dots, u_k$ in which nodes are added to S. Let $S_i = \{u_1, \dots, u_i\}$ be the set obtained after adding node u_i. It follows from line 38 in Algorithm A.2 that $E(u_{i+1}, S_i) \geq \gamma \|u_{i+1}\| \cdot \|S_i\|$ for $i = 1, \dots, k-1$. Furthermore, line 37 in Algorithm A.2 ensures that $\Delta\mathcal{H}(S_i \mapsto \emptyset) \leq 0$ for $i = 1, \dots, k-1$. We also have $\Delta\mathcal{H}(S_k \mapsto \emptyset) \leq 0$, since $S_k = S = v$ and since $\Delta\mathcal{H}(v \mapsto \emptyset) \leq 0$, as observed above. Taking into account that each u_i is subpartition γ-dense by our inductive hypothesis, this implies that each set S_i is subpartition γ-dense. Since $S = S_k$ is subpartition γ-dense, node v is subpartition γ-dense. Hence, each node $v \in V_\ell$ is subpartition γ-dense. This also holds for the nodes in the aggregate graph at the highest level in the Leiden algorithm, which implies that all communities in $\mathcal{P}_{t+1} = \mathcal{P}_t$ are subpartition γ-dense. In other words, $\mathcal{P}_{t+1} = \mathcal{P}_t$ is subpartition γ-dense. ■

Subpartition γ-density does not imply node optimality. It guarantees only that $\Delta\mathcal{H}(v \mapsto \emptyset) \leq 0$ for all $v \in V$, not that $\Delta\mathcal{H}(v \mapsto D) \leq 0$ for all $v \in V$ and all $D \in \mathcal{P}$. However, it is easy to see that all nodes are locally optimally assigned in a stable iteration of the Leiden algorithm. This is stated in the following theorem.

Theorem 7. Let $G = (V, E)$ be a graph, let $\mathcal{P}_t$ be a flat partition of G, and let $\mathcal{P}_{t+1} = \text{Leiden}(G, \mathcal{P}_t)$. If $\mathcal{P}_{t+1} = \mathcal{P}_t$, then $\mathcal{P}_{t+1} = \mathcal{P}_t$ is node optimal.

Proof. Suppose we have a stable iteration. Hence, $\mathcal{P}_{t+1} = \mathcal{P}_t$. We are going to give a proof by contradiction. Assume that $\mathcal{P}_{t+1} = \mathcal{P}_t$ is not node optimal. There then exists a node $v \in C \in \mathcal{P}_t$ and a community $D \in \mathcal{P}_t$ (or $D = \emptyset$) such that $\Delta\mathcal{H}(v \mapsto D) > 0$. The MoveNodesFast function then moves node v to community D. This means that $\mathcal{P}_{t+1} \neq \mathcal{P}_t$ and that the iteration is not stable. We now have a contradiction, which implies that the assumption of $\mathcal{P}_{t+1} = \mathcal{P}_t$ not being node optimal must be false. Hence, $\mathcal{P}_{t+1} = \mathcal{P}_t$ is node optimal. ■

In the same way, it is straightforward to see that the Louvain algorithm also guarantees node optimality in a stable iteration.

When the Louvain algorithm reaches a stable iteration, the partition is γ-separated and node optimal. Since the Louvain algorithm considers only moving nodes and merging communities, additional iterations of the algorithm will not lead to further improvements of the partition. Hence, in the case of the Louvain algorithm, if $\mathcal{P}_{t+1} = \mathcal{P}_t$, then $\mathcal{P}_\tau = \mathcal{P}_t$ for all $\tau \geq t$. In other words, when the Louvain algorithm reaches a stable iteration, all future iterations will be stable as well. This contrasts with the Leiden algorithm, which may continue to improve a partition after a stable iteration. We consider this in more detail below.

3. Asymptotic guarantees

When an iteration of the Leiden algorithm is stable, this does not imply that the next iteration will also be stable. Because of randomness in the refinement phase of the Leiden algorithm, a partition that is stable in one iteration may be improved in the next iteration. However, at some point, a partition will be obtained for which the Leiden algorithm is unable to make any further improvements. We call this an asymptotically stable partition. Below, we prove that an asymptotically stable partition is uniformly γ-dense and subset optimal.

We first need to show what it means to define asymptotic properties for the Leiden algorithm. The Leiden algorithm considers moving a node to a different community only if this results in a strict increase in the quality function. As stated in the following lemma, this ensures that at some point the Leiden algorithm will find a partition for which it can make no further improvements.

Lemma 8. Let $G = (V, E)$ be a graph, and let $\mathcal{P}_{t+1} = \text{Leiden}(G, \mathcal{P}_t)$. There exists a τ such that $\mathcal{P}_t = \mathcal{P}_\tau$ for all $t \geq \tau$.

Proof. Only strict improvements can be made in the Leiden algorithm. Consequently, if $\mathcal{P}_{t+1} \neq \mathcal{P}_t$, then $\mathcal{P}_{t+1} \neq \mathcal{P}_{t'}$ for all $t' \leq t$. Assume that there does not exist a τ such that $\mathcal{P}_t = \mathcal{P}_\tau$ for all $t \geq \tau$. Then for any τ there exists a $t > \tau$ such that $\mathcal{P}_t \neq \mathcal{P}_{t'}$ for all $t' < t$. This implies that the number of unique elements in the sequence $\mathcal{P}_0, \mathcal{P}_1, \dots$ is infinite. However, this is not possible, because the number of partitions of G is finite. Hence, the assumption that there does not exist a τ such that $\mathcal{P}_t = \mathcal{P}_\tau$ for all $t \geq \tau$ is false. ■

According to the above lemma, the Leiden algorithm progresses towards a partition for which no further improvements can be made. We can therefore define the notion of an asymptotically stable partition.

Definition 11. Let $G = (V, E)$ be a graph, and let $\mathcal{P}_{t+1} = \text{Leiden}(G, \mathcal{P}_t)$. We call $\mathcal{P}_\tau$ *asymptotically stable* if $\mathcal{P}_t = \mathcal{P}_\tau$ for all $t \geq \tau$.

We also need to define the notion of a minimal non-optimal subset.

Definition 12. Let $G = (V, E)$ be a graph, and let $\mathcal{P}$ be a partition of G. A set $S \subseteq C \in \mathcal{P}$ is called a *non-optimal subset* if $\Delta\mathcal{H}(S \mapsto D) > 0$ for some $D \in \mathcal{P}$ or for $D = \emptyset$. A set $S \subseteq C \in \mathcal{P}$ is called a *minimal non-optimal subset* if S is a non-optimal subset and if there does not exist a non-optimal subset $S' \subset S$.

The following lemma states an important property of minimal non-optimal subsets.

Lemma 9. Let $G = (V, E)$ be a graph, let $\mathcal{P}$ be a partition of G, and let $S \subseteq C \in \mathcal{P}$ be a minimal non-optimal subset. Then $\{S\}$ is an optimal partition of the subgraph induced by S.

Proof. Assume that $\{S\}$ is not an optimal partition of the subgraph induced by S. There then exists a set $S_1 \in S$ such that

$$E(S_1, S_2) - \gamma \|S_1\| \cdot \|S_2\| < 0, \tag{D2}$$

where $S_2 = S - S_1$. Let $D \in \mathcal{P}$ or $D = \emptyset$ such that $\Delta\mathcal{H}(S \to D) > 0$. Hence,

$$E(S, D) - \gamma \|S\| \cdot \|D\| > E(S, C - S) - \gamma \|S\| \cdot \|C - S\|. \tag{D3}$$

Because S is a minimal non-optimal subset, S_1 and S_2 cannot be non-optimal subsets. Therefore, $\Delta\mathcal{H}(S_1 \to D) \leq 0$ and $\Delta\mathcal{H}(S_2 \to D) \leq 0$, or equivalently,

$$E(S_1, D) - \gamma \|S_1\| \cdot \|D\| \leq E(S_1, C - S_1) - \gamma \|S_1\| \cdot \|C - S_1\| \tag{D4}$$

and

$$E(S_2, D) - \gamma \|S_2\| \cdot \|D\| \leq E(S_2, C - S_2) - \gamma \|S_2\| \cdot \|C - S_2\|. \tag{D5}$$

It then follows from Eqs. (D4) and (D5) that

$$\begin{aligned} E(S, D) - \gamma \|S\| \cdot \|D\| &= \big(E(S_1, D) - \gamma \|S_1\| \cdot \|D\|\big) + \big(E(S_2, D) - \gamma \|S_2\| \cdot \|D\|\big) \\ &\leq \big(E(S_1, C - S_1) - \gamma \|S_1\| \cdot \|C - S_1\|\big) + \big(E(S_2, C - S_2) - \gamma \|S_2\| \cdot \|C - S_2\|\big). \end{aligned}$$

This can be written as

$$\begin{aligned} E(S, D) - \gamma \|S\| \cdot \|D\| \leq \; & \big(E(S_1, C - S) + E(S_1, S_2) - \gamma \|S_1\| \cdot \|C - S\| - \gamma \|S_1\| \cdot \|S_2\|\big) \\ & + \big(E(S_2, C - S) + E(S_2, S_1) - \gamma \|S_2\| \cdot \|C - S\| - \gamma \|S_2\| \cdot \|S_1\|\big) \\ = \; & E(S, C - S) + 2E(S_1, S_2) - \gamma \|S\| \cdot \|C - S\| - 2\gamma \|S_1\| \cdot \|S_2\|. \end{aligned}$$

Using Eq. (D2), we then obtain

$$E(S, D) - \gamma \|S\| \cdot \|D\| < E(S, C - S) - \gamma \|S\| \cdot \|C - S\|.$$

However, this contradicts Eq. (D3). The assumption that $\{S\}$ is not an optimal partition of the subgraph induced by S is therefore false. ■

Building on the results for non-decreasing move sequences reported in Appendix C 1, the following lemma states that any minimal non-optimal subset can be found by the MergeNodeSubset function.

Lemma 10. Let $G = (V, E)$ be a graph, let $\mathcal{P}$ be a partition of G, and let $S \subseteq C \in \mathcal{P}$ be a minimal non-optimal subset. Let $\mathcal{P}_{\text{refined}} = \text{MergeNodesSubset}(G, \{\{v\} \mid v \in V\}, C)$. There then exists a move sequence in the MergeNodesSubset function such that $S \in \mathcal{P}_{\text{refined}}$.

Proof. We are going to prove that there exists a move sequence $\mathcal{P}_0, \ldots, \mathcal{P}_{|C|}$ in the MergeNodesSubset function such that $S \in \mathcal{P}_{|C|}$. The move sequence consists of two parts, $\mathcal{P}_0, \ldots, \mathcal{P}_{|S|}$ and $\mathcal{P}_{|S|}, \ldots, \mathcal{P}_{|C|}$. In the first part, each node in S is considered for moving. In the second part, each node in $C - S$ is considered for moving. Note that in the MergeNodesSubset function a node can always stay in its own community when it is considered for moving. We first consider the first part of the move sequence $\mathcal{P}_0, \ldots, \mathcal{P}_{|C|}$. Let $\mathcal{P}_0, \ldots, \mathcal{P}_{|S|}$ be a non-decreasing move sequence such that $\mathcal{P}_0 = \{\{v\} \mid v \in V\}$ and $S \in \mathcal{P}_{|S|}$. To see that such a non-decreasing move sequence exists, note that according to Lemma 9 $\{S\}$ is an optimal partition of the subgraph induced by S and that according to Theorem 1 an optimal partition can be reached using a non-decreasing move sequence. This non-decreasing move sequence consists of $|S| - 1$ moves. There is one node in S that can stay in its own community. Note further that each move in the move sequence $\mathcal{P}_0, \ldots, \mathcal{P}_{|S|}$ satisfies the conditions specified in lines 34 and 37 in Algorithm A.2. This follows from Definition 12. In the second part of the move sequence $\mathcal{P}_0, \ldots, \mathcal{P}_{|C|}$, we simply have $\mathcal{P}_{|S|} = \ldots = \mathcal{P}_{|C|}$. Hence, each node in $C - S$ stays in its own community. Since $S \in \mathcal{P}_{|S|}$, we then also have $S \in \mathcal{P}_{|C|}$. ■

As long as there are subsets of communities that are not optimally assigned, the MergeNodesSubset function can find these subsets. In the MoveNodesFast function, these subsets are then moved to a different community. In this way, the Leiden algorithm continues to identify better partitions. However, at some point, all subsets of communities are optimally assigned, and the Leiden algorithm will not be able to further improve the partition. The algorithm has then reached an asymptotically stable partition, and this partition is also subset optimal. This result is formalized in the following theorem.

Theorem 11. Let $G = (V, E)$ be a graph, and let $\mathcal{P}$ be a flat partition of G. Then $\mathcal{P}$ is asymptotically stable if and only if $\mathcal{P}$ is subset optimal.

Proof. If $\mathcal{P}$ is subset optimal, it follows directly from the definition of the Leiden algorithm that $\mathcal{P}$ is asymptotically stable. Conversely, if $\mathcal{P}$ is asymptotically stable, it follows from Lemma 10 that $\mathcal{P}$ is subset optimal. To see this, assume that $\mathcal{P}$ is not subset optimal. There then exists a community $C \in \mathcal{P}$ and a set $S \subset C$ such that S is a minimal non-optimal subset. Let $\mathcal{P}_{\text{refined}} = \text{MergeNodesSubset}(G, \{\{v\} \mid v \in V\}, C)$. Lemma 10 states that there exists a move sequence in the MergeNodesSubset function such that $S \in \mathcal{P}_{\text{refined}}$. If $S \in \mathcal{P}_{\text{refined}}$, then S will be moved from C to a different (possibly empty) community in line 3 in Algorithm A.2. However, this contradicts the asymptotic stability of $\mathcal{P}$. Asymptotic stability therefore implies subset optimality. ■

Since subset optimality implies uniform γ-density, we obtain the following corollary.

Corollary 12. Let $G = (V, E)$ be a graph, and let $\mathcal{P}$ be a flat partition of G. If $\mathcal{P}$ is asymptotically stable, then $\mathcal{P}$ is uniformly γ-dense.

Appendix E: Bounds on optimality

In this appendix, we prove that the quality of a uniformly γ-dense partition as defined in Definition 9 in Appendix D provides an upper bound on the quality of an optimal partition.

We first define the intersection of two partitions.

Definition 13. Let $G = (V, E)$ be a graph, and let $\mathcal{P}_1$ and $\mathcal{P}_2$ be flat partitions of G. We denote the *intersection* of $\mathcal{P}_1$ and $\mathcal{P}_2$ by $\mathcal{P} = \mathcal{P}_1 \sqcap \mathcal{P}_2$, which is defined as

$$\mathcal{P} = \{C \cap D \mid C \in \mathcal{P}_1, D \in \mathcal{P}_2, C \cap D \neq \emptyset\}. \tag{E1}$$

The intersection of two partitions consists of the basic subsets that form both partitions. For $S, R \in \mathcal{P} = \mathcal{P}_1 \sqcap \mathcal{P}_2$, we write $S \overset{\mathcal{P}_1}{\sim} R$ if there exists a community $C \in \mathcal{P}_1$ such that $S, R \subseteq C$. Hence, if $S \overset{\mathcal{P}_1}{\sim} R$, then S and R are subsets of the same community in $\mathcal{P}_1$. Furthermore, for $S \neq R$, if $S \overset{\mathcal{P}_1}{\sim} R$, then we cannot have $S \overset{\mathcal{P}_2}{\sim} R$, since otherwise S and R would have formed a single subset. In other words, $S \overset{\mathcal{P}_1}{\sim} R \Rightarrow S \overset{\mathcal{P}_2}{\nsim} R$ and similarly $S \overset{\mathcal{P}_2}{\sim} R \Rightarrow S \overset{\mathcal{P}_1}{\nsim} R$.

The following lemma shows how the difference in quality between two partitions can easily be expressed using the intersection.

Lemma 13. Let $G = (V, E)$ be a graph, let $\mathcal{P}_1$ and $\mathcal{P}_2$ be flat partitions of G, and let $\mathcal{P} = \mathcal{P}_1 \sqcap \mathcal{P}_2$ be the intersection of $\mathcal{P}_1$ and $\mathcal{P}_2$. Then

$$\mathcal{H}(\mathcal{P}_2) - \mathcal{H}(\mathcal{P}_1) = \frac{1}{2} \sum_{\substack{S \overset{\mathcal{P}_2}{\sim} R \\ S \neq R}} \left[E(S, R) - \gamma \|S\| \cdot \|R\|\right] - \frac{1}{2} \sum_{\substack{S \overset{\mathcal{P}_1}{\sim} R \\ S \neq R}} \left[E(S, R) - \gamma \|S\| \cdot \|R\|\right]. \tag{E2}$$

Proof. For any community $C \in \mathcal{P}_k$ $(k = 1, 2)$,

$$E(C, C) = \sum_{\substack{S \in \mathcal{P} \\ S \subseteq C}} E(S, S) + \frac{1}{2} \sum_{\substack{S, R \in \mathcal{P} \\ S, R \subseteq C \\ S \neq R}} E(S, R)$$

and

$$\binom{\|C\|}{2} = \sum_{\substack{S \in \mathcal{P} \\ S \subseteq C}} \binom{\|S\|}{2} + \frac{1}{2} \sum_{\substack{S, R \in \mathcal{P} \\ S, R \subseteq C \\ S \neq R}} \|S\| \cdot \|R\|.$$

We hence obtain

$$\begin{aligned}\mathcal{H}(\mathcal{P}_k) &= \sum_{C \in \mathcal{P}_k} \left[E(C,C) - \gamma \binom{\|C\|}{2} \right] \\ &= \sum_{C \in \mathcal{P}_k} \left[\sum_{\substack{S \in \mathcal{P} \\ S \subseteq C}} \left[E(S,S) - \gamma \binom{\|S\|}{2} \right] + \frac{1}{2} \sum_{\substack{S,R \in \mathcal{P} \\ S,R \subseteq C \\ S \neq R}} \left[E(S,R) - \gamma \|S\| \cdot \|R\| \right] \right] \\ &= \sum_{S \in \mathcal{P}} \left[E(S,S) - \gamma \binom{\|S\|}{2} \right] + \frac{1}{2} \sum_{\substack{S \overset{\mathcal{P}_k}{\sim} R \\ S \neq R}} \left[E(S,R) - \gamma \|S\| \cdot \|R\| \right].\end{aligned}$$

The difference $\mathcal{H}(\mathcal{P}_2) - \mathcal{H}(\mathcal{P}_1)$ then gives the desired result. ■

The above lemma enables us to prove the following theorem, stating that the quality of a uniformly γ-dense partition is not too far from optimal. We stress that this theorem applies only to unweighted graphs.

Theorem 14. Let $G = (V, E)$ be an unweighted graph, let $\mathcal{P}$ be a uniformly γ-dense partition of G, and let $\mathcal{P}^*$ be an optimal partition of G. Then

$$\mathcal{H}(\mathcal{P}^*) - \mathcal{H}(\mathcal{P}) \leq (1-\gamma) \frac{1}{2} \sum_{\substack{C,D \in \mathcal{P} \\ C \neq D}} E(C,D). \tag{E3}$$

Proof. Let $\mathcal{P}' = \mathcal{P} \sqcap \mathcal{P}^*$. Consider any $S, R \in \mathcal{P}'$ such that $S \overset{\mathcal{P}^*}{\sim} R$. Because the graph G is unweighted, we have $\|S\| \cdot \|R\| \geq E(S,R)$. It follows that

$$E(S,R) - \gamma \|S\| \cdot \|R\| \leq (1-\gamma) E(S,R).$$

Furthermore, for any community $C \in \mathcal{P}$, the number of edges connecting this community with other communities is $E(C, V - C)$. We therefore have

$$\sum_{S \subseteq C} \sum_{\substack{R \overset{\mathcal{P}^*}{\sim} S \\ R \neq S}} E(S,R) \leq E(C, V - C).$$

To see this, note that $R \overset{\mathcal{P}^*}{\sim} S$ implies $R \overset{\mathcal{P}}{\nsim} S$, so that $R \nsubseteq C$. For any $C \in \mathcal{P}$, we then obtain

$$\sum_{S \subseteq C} \sum_{\substack{R \overset{\mathcal{P}^*}{\sim} S \\ R \neq S}} \left[E(S,R) - \gamma \|S\| \cdot \|R\| \right] \leq \sum_{S \subseteq C} \sum_{\substack{R \overset{\mathcal{P}^*}{\sim} S \\ R \neq S}} (1-\gamma) E(S,R) \leq (1-\gamma) E(C, V - C).$$

By summing over all $C \in \mathcal{P}$, this gives

$$\sum_{\substack{S \overset{\mathcal{P}^*}{\sim} R \\ S \neq R}} \left[E(S,R) - \gamma \|S\| \cdot \|R\| \right] \leq (1-\gamma) \sum_{\substack{C,D \in \mathcal{P} \\ C \neq D}} E(C,D).$$

Furthermore, because $\mathcal{P}$ is uniformly γ-dense, we have

$$\sum_{\substack{S \overset{\mathcal{P}}{\sim} R \\ S \neq R}} \left[E(S,R) - \gamma \|S\| \cdot \|R\| \right] \geq 0.$$

Using these results, Eq. (E3) follows from Lemma 13. ■

For weighted graphs, an upper bound analogous to Eq. (E3) is

$$\mathcal{H}(\mathcal{P}^*) - \mathcal{H}(\mathcal{P}) \leq \left(1 - \frac{\gamma}{\bar{w}}\right) \frac{1}{2} \sum_{C,D\in\mathcal{P}} E(C,D), \tag{E4}$$

where $\bar{w} = \max_{i,j} w_{i,j}$ is the maximum edge weight.

For modularity instead of CPM, the upper bound for unweighted graphs in Eq. (E3) needs to be adjusted by rescaling the resolution parameter by $2m$. This gives

$$\mathcal{H}(\mathcal{P}^*) - \mathcal{H}(\mathcal{P}) \leq \left(1 - \frac{\gamma}{2m}\right) \frac{1}{2} \sum_{\substack{C,D\in\mathcal{P}\\ C\neq D}} E(C,D). \tag{E5}$$

The approximation factor of modularity cannot be multiplicative [7], and indeed our bound is additive. Depending on the partition $\mathcal{P}$, our bound may be better than the bound provided by an SDP algorithm [7].

Note that the bound in Eq. (E3) reduces the trivial bound of $(1-\gamma)m$ by γ times the number of missing links within communities, i.e., $\gamma \sum_C \left[\binom{\|C\|}{2} - E(C,C)\right]$. To see this, note that $m = \sum_C E(C,C) + \frac{1}{2}\sum_{C\neq D} E(C,D)$. Starting from Eq. (E3), we then obtain

$$\begin{aligned}
\mathcal{H}(\mathcal{P}^*) &\leq \mathcal{H}(\mathcal{P}) + (1-\gamma)\frac{1}{2} \sum_{\substack{C,D\in\mathcal{P}\\ C\neq D}} E(C,D) \\
&= \sum_{C\in\mathcal{P}} \left[E(C,C) - \gamma\binom{\|C\|}{2}\right] + (1-\gamma)m - (1-\gamma)\sum_{C\in\mathcal{P}} E(C,C) \\
&= (1-\gamma)m - \gamma \sum_{C\in\mathcal{P}} \left[\binom{\|C\|}{2} - E(C,C)\right].
\end{aligned}$$

Finally, Theorem 14 provides a bound on the quality of the optimal partition for a given uniformly γ-dense partition, but it does not provide an a priori bound on the minimal quality of a uniformly γ-dense partition. Finding such an a priori bound remains an open problem.

[1] V. A. Traag and J. Bruggeman, Phys. Rev. E **80**, 036115 (2009).
[2] V. A. Traag, P. Van Dooren, and Y. Nesterov, Phys. Rev. E **84**, 016114 (2011).
[3] S. Fortunato and M. Barthélemy, Proc. Natl. Acad. Sci. U. S. A. **104**, 36 (2007).
[4] M. E. J. Newman and M. Girvan, Phys. Rev. E **69**, 026113 (2004).
[5] R. Guimerà and L. A. Nunes Amaral, Nature **433**, 895 (2005).
[6] J. Reichardt and S. Bornholdt, Phys. Rev. E **74**, 016110 (2006).
[7] T. N. Dinh, X. Li, and M. T. Thai, in *2015 IEEE Int. Conf. Data Min.* (IEEE, 2015) pp. 101–110.
